# Universal clinical Parkinson’s disease axes identify a major influence of neuroinflammation

**DOI:** 10.1186/s13073-022-01132-9

**Published:** 2022-11-16

**Authors:** Cynthia Sandor, Stephanie Millin, Andrew Dahl, Ann-Kathrin Schalkamp, Michael Lawton, Leon Hubbard, Nabila Rahman, Nigel Williams, Yoav Ben-Shlomo, Donald G. Grosset, Michele T. Hu, Jonathan Marchini, Caleb Webber

**Affiliations:** 1 UK Dementia Research Institute, Cardiff University, Cardiff, CF24 4HQ UK; 2 Department of Physiology, Anatomy and Genetics, University of Oxford, Oxford, OX1 3PT UK; 3 Wellcome Centre for Human Genetics, University of Oxford, Oxford, OX3 7BN UK; 4 School of Social and Community Medicine, University of Bristol, Bristol, BS8 1TH UK; 5 MRC Centre for Neuropsychiatric Genetics and Genomics, Institute of Psychological Medicine and Clinical Neurosciences, School of Medicine, Cardiff University, Cardiff, CF24 4HQ UK; 6 Department of Neurology, Institute of Neurological Sciences, Queen Elizabeth University Hospital, G51 4LB Glasgow, UK; 7 Department of Physiology, Anatomy and Genetics, Le Gros Clark Building, Oxford Parkinson’s Disease Centre, University of Oxford, Oxford, OX1 3PT UK; 8 Nuffield Department of Clinical Neurosciences, Division of Clinical Neurology, University of Oxford, Oxford, OX3 7LF UK; 9 Department of Statistics, University of Oxford, Oxford, OX1 UK; 10 Regeneron Genetics Center, Tarrytown, NY USA

**Keywords:** Parkinson’s disease, Deeply phenotyped cohorts, Neuro-inflammation

## Abstract

**Background:**

There is large individual variation in both clinical presentation and progression between Parkinson’s disease patients. Generation of deeply and longitudinally phenotyped patient cohorts has enormous potential to identify disease subtypes for prognosis and therapeutic targeting.

**Methods:**

Replicating across three large Parkinson’s cohorts (Oxford Discovery cohort (*n* = 842)/Tracking UK Parkinson’s study (*n* = 1807) and Parkinson’s Progression Markers Initiative (*n* = 472)) with clinical observational measures collected longitudinally over 5–10 years, we developed a Bayesian multiple phenotypes mixed model incorporating genetic relationships between individuals able to explain many diverse clinical measurements as a smaller number of continuous underlying factors (“phenotypic axes”).

**Results:**

When applied to disease severity at diagnosis, the most influential of three phenotypic axes “Axis 1” was characterised by severe non-tremor motor phenotype, anxiety and depression at diagnosis, accompanied by faster progression in cognitive function measures. Axis 1 was associated with increased genetic risk of Alzheimer’s disease and reduced CSF Aβ1-42 levels. As observed previously for Alzheimer’s disease genetic risk, and in contrast to Parkinson’s disease genetic risk, the loci influencing Axis 1 were associated with microglia-expressed genes implicating neuroinflammation. When applied to measures of disease progression for each individual, integration of Alzheimer’s disease genetic loci haplotypes improved the accuracy of progression modelling, while integrating Parkinson’s disease genetics did not.

**Conclusions:**

We identify universal axes of Parkinson’s disease phenotypic variation which reveal that Parkinson’s patients with high concomitant genetic risk for Alzheimer’s disease are more likely to present with severe motor and non-motor features at baseline and progress more rapidly to early dementia.

**Supplementary Information:**

The online version contains supplementary material available at 10.1186/s13073-022-01132-9.

## Background

A critical challenge in medicine is to understand why the clinical presentations of each patient affected by the same disorder vary. This is especially true for Parkinson’s disease, for which the age of onset, the rate of progression, type and severity of symptoms differ across more than a million people worldwide living with this disease [1]. To accelerate the identification of disease subtypes, large deeply phenotyped cohorts of Parkinson’s disease patients have been created, in which valuable clinical, imaging, biosample and genetic data have been collected, increasingly with longitudinal monitoring [2–4].

Recent studies exploiting these deeply phenotyped cohorts have classified patients into discrete phenotypic subgroups, each displaying a characteristic set of symptoms [5–7]. To define Parkinson’s disease subtypes, most of these studies employ some form of variable selection to create a distance matrix between individuals, followed by clustering methods such as k-means or hierarchical clustering. These methods provide discrete phenotypic groups, which are appealing in their categorical nature but have many shortfalls. Firstly, while selection methods quantify how much variance each phenotype explains, no robust method has defined a threshold for this measure above which a phenotype contributes to the distance matrix. Consequently, the definition of which phenotypes are essential to classify patients, and which are irrelevant can be somewhat arbitrary. For example, two recent studies [5, 8], using the same Parkinson’s Progression Markers Initiative (*PPMI*) cohort show divergent results: apathy and hallucinations were key subtype classifiers in the first study [8], but not in the second one [5], because these variables were not included. Secondly, K-means clustering requires the number of phenotypic groups to be prespecified, and this choice has the potential to be biased towards preconceived expectations with smaller groups ignored or erroneously joined with larger groups. Two studies using a k-means approach and the same cohort came to different conclusions. Lawton et al. (2015) [6] and Lawton et al. (2018) [7] identified five and four clusters, respectively, with some individuals previously in the same cluster moving to different clusters. This discrepancy reflects that the optimal number of clusters is not trivial to select and different statistics used to decide on optimal numbers often disagree. Finally, the creation of discrete groups may not reflect the possibly continuous nature of phenotypic variability and ignores the greater statistical power of continuous traits.

To overcome these limitations, we propose here an approach focused on the continuous variation of phenotypes. For this, we applied PHENIX (PHENotype Imputation eXpediated), a multiple phenotype mixed model (MPMM) approach initially developed to impute missing phenotypes [9], that is employed here to perform genetically-guided dimensionality reduction of multiple clinical traits. This approach models the phenotypes as a combination of genetic and environmental factors, and the genetic component exploits the genetic relatedness between patients.

Applying PHENIX to the deeply phenotyped UK-based *Oxford Discovery* cohort [4, 6], we identify a small number of axes underlying individual Parkinson’s disease patient phenotypic variation that explain the variation in the much larger number of clinically-observed phenotypes. We demonstrate the universality of these axes of phenotypic variation amongst Parkinson’s disease patients by independently deriving similar axes in all three deeply phenotyped cohorts, namely *Tracking UK* cohort [2], the UK *Oxford Discovery* cohort [4, 6] and lastly the US *Parkinson’s Progression Markers Initiative *(PPMI) cohort that has a different clinical structure from the UK cohorts. We show that this reproducibility is not achieved by other commonly-used dimensionality-reduction methods and the utility of a genetic component. Finally, we demonstrate that the most influential phenotypic axis was associated with the genetic risk of Alzheimer’s disease and microglia-specific gene expression, suggesting Parkinson’s disease patients with a high genetic risk for Alzheimer’s disease are more likely to develop an aggressive form of Parkinson’s disease including dementia symptoms.

## Methods

### Clinical cohorts

#### Oxford Discovery cohort

We considered 842 Parkinson’s disease cases from the *Oxford Discovery* cohort [4, 6]. Individuals were required to have at least 90% chance of Parkinson’s disease according to UK-Parkinson’s disease brain bank criteria, no alternative diagnosis and disease duration less than 3.5 years. All patients had a clinical assessment repeated every eighteen months and have been already described [4, 6]. Phenotype data were collected for over a hundred clinical attributes, affecting autonomic, neurological and motor phenotypes (Additional file 1: Fig S1) and described in the Additional file 2: Table S1. Genotype data were generated using the Illumina HumanCoreExome-12 v1.1 and Illumina InfiniumCoreExome-24 v1.1 SNP arrays. To access to the clinical data of the *Oxford Discovery* cohort [4, 6], researchers must apply to the Oxford Parkinson’s Disease Centre (OPDC).

#### Tracking UK Parkinson’s study

We considered 1807 Parkinson’s disease cases from the *Tracking UK* Parkinson’s cohort, which was already described in detail by Malek et al. [2] Genotype data were generated using the Illumina Human Core Exome array. To access to the clinical data of the *Tracking UK* cohort, researchers must contact Dr Donal Grosset (donaldgrosset@gmail.com).

### Parkinson’s Progression Markers Initiative cohort

The *PPMI* cohort (http://www.ppmi-info.org) was already described in detail (including the *PPMI* protocol of recruitment and informed consent) by Marrek et al*.* [10]. We downloaded data from the *PPMI* database on January 2021 in compliance with the *PPMI* Data Use Agreement. We considered 472 newly-diagnosed Parkinson’s disease subjects: subjects with a diagnosis of Parkinson’s disease for two years or less and who are not taking Parkinson’s disease medications. We used the baseline (*t* = 0) (Additional file 2: Table S2) and the follow-up of clinical assessments. We excluded any individual with > 5% of missing data (437 individuals included). Participants have been genotyped using the NeuroX chip [11, 12]. *PPMI* data are available to the research community on the *PPMI* website: www.ppmi-info.org.

### Clinical score of severity at diagnosis and progression

The major difference between both UK cohorts and the PPMI cohort [10] is that the UK patients are older and already under medication during the recruitment (Table 1).

**Table 1:** Comparison of the patient’s clinical profile in the three cohorts at recruitment

	Cohort					
	Discovery (*N* = 876)		Tracking (*N* = 1725)		PPMI (*N*=472)	
	Mean	Std	Mean	Std	Mean	Std
Under medication (fraction)	0.87	0.34	0.9	0.3	0.06	0.24
UPDRS I	8.75	5.02	9.21	5.35	6	4.6
UPDRS II	8.66	5.96	9.72	6.48	6.46	4.66
UPDRS III	26.46	11.3	22.85	12.3	20.74	9.72
Age (years)	67.17	9.31	67.21	9.08	61.47	9.82
Age at diagnostic (years)	65.93	9.35	65.87	9.07	61.02	9.8
Disease duration (years)	1.24	0.94	1.34	0.9	0.76	0.57
Number of clinical visits	3.61	1.17	3.59	1.04	11.14	3.58
Male (fraction)	0.64	0.48	0.65	0.48	0.65	0.48

As the clinical measures can be confounded by differences in disease stage and medication status, an estimate of the disease severity at diagnosis as well as a measure of disease progression for each individual was derived with linear mixed effect models (LMM) by adjusting for different covariates. LMMs were chosen as they handle longitudinal data, i.e. non-independent data, allow for missing values and a flexible modelling of time, and can estimate individual trends [13]. For *PPMI* [10], *Oxford Discovery* [4, 6] and *Tracking UK * [2]*.* longitudinal data for several clinical tests was available (Tables S1 and S2). The clinical tests are recoded such that higher values indicate worse performance (multiplied by − 1) and standardised. For each clinical test, we fitted a LMM. We consider an intercept and the time since diagnosis as random effects such that for each individual we get an estimate for the severity at diagnosis and the progression respectively. We further included sex (categorical), education years (standardised), and age at diagnosis (standardised) as fixed effects. Our final model can be described as: clinical_assessment ~ 1 + C(sex) + education_years + age_at_diagnosis + time_since_diagnosis + (1 + time_since_diagnosis | subject_id). In both cohorts, clinical tests were performed both medicated and not medicated (Additional file 1: Fig S2). We therefore include an additional fixed effect indicating medication usage (categorical). Inclusion criteria are having less than 5% of missing data and at least two visits for a clinical test (decided for each clinical test individually) and having no missing information for the random and fixed effects. For *PPMI* 472 subjects with a median of 12 visits spanning over a mean of 5.59 years after diagnosis are included and for *Oxford Discovery* 876 subjects with a median of 4 visits spanning over a mean of 4.38 years after diagnosis are included. The goodness of fit was estimated as the R^2^ (e.g. for UPDRS III Additional file 1: Fig S3 or Additional file 2: Table S3). We noted that are not perfectly normally distributed as illustrated in Additional file 1: Fig S4. Nevertheless, we did not observe a significant improvement of goodness after box-blot data transformation so that it meets the assumption of normality. While we applied linear mixed models here, we acknowledge the current debate over whether linear or non-linear mixed models best model the data [14, 15]. Individual estimates for disease severity at diagnosis and disease progression can be extracted from the random effects. These measures are used for further analyses. The LMMs were fitted with pymer4 0.7.1and the model comparison was done with scikit-learn 0.23.2.

### Genotype: quality control and imputation

Quality control was carried out independently using PLINK v1.9 [16]. Imputation of unobserved and missing variants was carried out separately for each cohort (Supplemental Material).

### Phenotypic axis

**Fig. 1:**
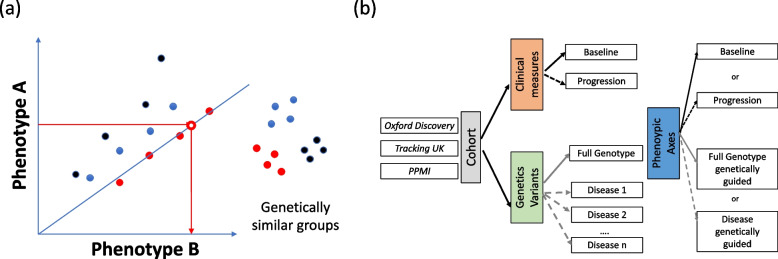
Identifying the underlying phenotypic axes using a Bayesian Mixed Model that incorporates genetic similarity. **a** Schematic representation of the approach used to capture the latent axes of clinical variation in deeply phenotyped cohorts. The method (PHENIX) was initially developed to impute missing observations (e.g. phenotype A) according to other available observations (here B). This approach also exploits genetic relationships where phenotypic heritability can be used to increase imputation accuracy. Here, we identify the relationship (diagonal blue) derived by PHENIX, herein named phenotypic axis, to capture the clinical variation. **b** Workflow of the analyses performed here. In each of the three cohorts, we independently derived the phenotypic axes associated with the baseline clinical variation and the phenotypic axes associated with the clinical progression. We also derived the phenotypic axes by exploiting the full genotype available or, instead by selecting the genotype in a subset of loci associated with a specific disorder

Our continuous measures of severity are based on a multiple phenotypes mixed model (MPMM) approach named PHENIX (PHENotype Imputation eXpediated) which includes genetic relationships between individuals and was designed to impute missing phenotypes [9]. To impute missing phenotypes, PHENIX reduces the variation within a cohort to a smaller number of underlying factors that are then used to predict individual missing values. Here, we exploit the identification of these underlying factors as providing the latent axes of patient variation which underlie a larger number of clinically observed phenotypes (Fig. 1A). The outcome is that the many clinical phenotypes (sometimes missing for some individuals) of each individual are represented through, i.e. their variances may be well explained by, a smaller number of underlying latent variables of phenotypic variation, which we name herein as *phenotypic axes*.

PHENIX [9] employs a Bayesian multiple-phenotype mixed model (MPMM), where the correlations between clinical phenotypes (*Y*) are decomposed into a genetic and a residual component with the following model: *Y* = *U* + *e*, where *U* represents the aggregate genetic contribution (whole genotype) to phenotypic variance and *e* is idiosyncratic noise. As the estimation of maximum likelihood covariance estimates can become computationally expensive with an increasing number of phenotypes, PHENIX uses a Bayesian low-rank matrix factorization model for the genetic term *U* such that: *U* = Sβ, in which β is can be used to estimate the genetic covariance matrix between phenotypes and S represents a matrix of latent components that each follow ~ N (0,G) where G is the Estimate of Relatedness Matrix from genotypes. The resulting latent traits (S) are used here as phenotypic axes, each representing the severity of a number of non-independent clinical phenotypes. The details to run PHENIX and extract the phenotypic axes are given in the Supplemental Material.

### Risk-guided phenotypic axis

We derived a risk-guided phenotypic axis by replacing the whole-genotype-relatedness genetic component in our MPMM by the genetic relatedness focused upon a specific disease/trait (Fig. 1B). To calculate a disease-relatedness matrix, we recalculated relatedness between individuals using only those genetic variants (after pruning) with a genome-wide association study (GWAS) association < 0.05, and repeated at < 0.1, with a given human complex trait. For different complex human traits with GWAS results publicly available (Additional file 2:Table S4), we calculated a disease relatedness genotypic similarity matrix between patients that we used subsequently to derive phenotypic axes (Additional file 1) We determined statistical significance associated with any increase in the phenotypic variation explained by the new, risk-guided, phenotypic axes by deriving phenotypes with random SNPs sets matching the number of SNPs (after pruning) with *p*-value < 0.05 (or < 0.01). We calculated an empirical p-value by comparing the phenotypic variation explained by the risk-guided, phenotypic axes with the phenotypic variation explained by random SNPs’ phenotypic axes.

### Conditional risk-guided phenotypic axis type association analysis

To evaluate whether the subset of genetic variants associated with a specific disease that influences a phenotypic axis overlap with those influencing another disease, we performed GWA conditional analysis with multi-trait-based conditional and joint analysis (mtCOJO) [17]. We recalculated the genetic similarity with the summary GWA statistic of a trait conditioned for those of another trait and derived the new conditional risk-guided phenotypic axis. After this conditional analysis, we then examined whether the proportion of the phenotypic variance explained decreased or not: a decreasing proportion suggests that overlapping genetic variants of the two traits were associated with the same phenotypic axis.

### Cell type association analysis

With the same approach and dataset described by Agarwal et al. (2020) [18], we examined the intersection between Substantia nigra (SN) cell type-specific gene expression patterns and the genetics influencing the phenotypic axes to identify disease-relevant cell types in the brain. We performed these cell type association analyses using MAGMA [19].

### Microglia-specific module analysis

We used the same approach and the same dataset as Agarwal et al. (2020) [18]. Briefly, a microglia-specific protein–protein interaction (PPI) network is built by identifying PPIs between genes highly expressed in the SN microglia. We then identified modules of highly interconnected genes in a microglia type-specific PPI network using the “cluster_louvain” function in “igraph” R package [20]. To functional annotate each module, we performed Gene Ontology (GO) enrichment analysis with topGO R Bioconductor package [21] by testing the over-representation of GO biological processes (GO BP) terms within the module gene sets using Fisher’s test. rrvgo R Bioconductor package [22] was used to summarise the top 100 enriched GO BP terms into a smaller number of representative terms.

## Results

### Three continuous measures capture 75% of the clinical variation

**Fig. 2:**
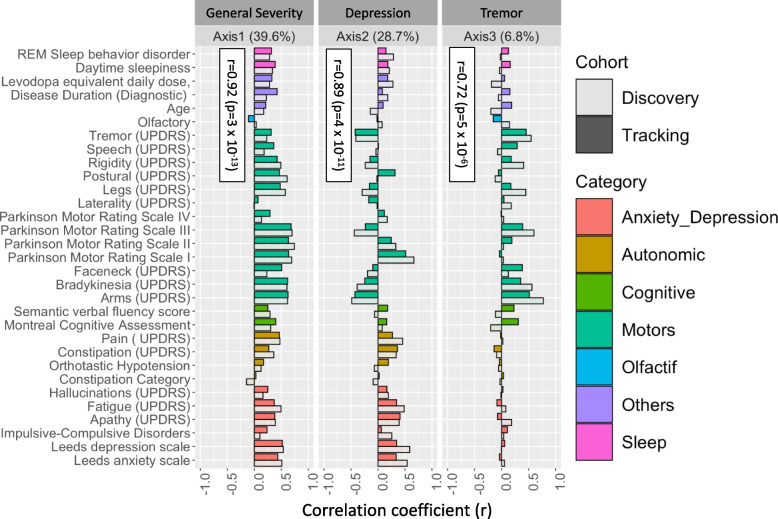
Similar phenotypic axes are obtained in two deeply phenotyped Parkinson’s disease cohorts. Results were consistent in two independent cohorts (842 O*xford Discovery* and 1807 *Tracking UK* patients). Examination of these two separate Parkinson’s disease cohorts, using an independent derivation of the phenotypic axes in each, showed significant correlations between each cohort’s first three axes. Correlations between the axes from each cohort are Axis 1 *r* = 0.92 (*p* = 3 × 10^–13^), Axis 2 *r* = 0.89 (*p* = 4 × 10^−11^), and Axis 3 *r* = 0.72 (*p* = 5 × 10^*−*6^). The correlation coefficient (*x*-axis) between each axis derived in each cohort (light: *Oxford Discovery* vs dark: *Tracking UK*) and each clinical observation (*y*-axis) is shown. We represented six major categories of Parkinson’s disease symptoms by the colour of the bar plots. These categories include anxiety and depression, the autonomic system, cognitive functions, the motor system, the olfactory system and sleep disorders. The Unified Parkinson’s Disease Rating Scale (UPDRS) is a comprehensive 50-question assessment of both motor and non-motor symptoms associated with Parkinson’s. It includes four parts: (I) non-motor experiences of daily living (II) motor experiences of daily living (III) motor examination (IV) motor complications

**Fig. 3:**
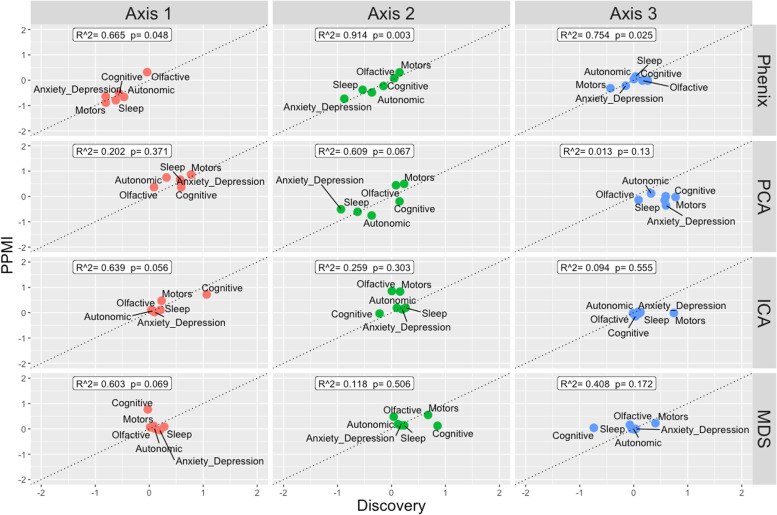
Other methods fail to align between different but deeply phenotyped UK and US Parkinson’s disease cohorts. We compared the ability of different dimensionality reduction methods (independent component analysis (ICA), multidimensional scaling (MDS), principal component analysis (PCA) and phenotypic axis based on the PHENIX multiple phenotype mixed model) to phenotypically align two deeply phenotyped Parkinson’s disease cohorts, specifically the *Oxford Discovery* (842 individuals) and *PPMI* (439 sporadic Parkinson’s disease) cohorts. The *x*-axis and *y*-axis represent the correlation coefficient between each continuous variable with clinical observation associated with a specific symptom category in *Oxford Discovery* and *PPMI* cohort, respectively. Each column panel and colour of points (“Axis”) represents the dimension level of each underlying dimension. All points on the diagonal would represent a perfect phenotypic alignment of both cohorts. We examined the relationship between correlation derived from both cohorts by performing a linear regression: *R*^*^2*^ and p correspond to the coefficient of determination and the *p*-value respectively. Only the dimensions discovered by the MPMM model, PHENIX, show a significant relationship between both cohorts: MPMM phenotypic axes (*R*^2^ = 0.86, *p* = 2 × 10^−8^), MDS (*R*^*2*^ = 0.11, *p* = 0.18), ICA (*R*^2^ = 0.17, *p* = 0.16) and PCA (*R*.^2^ = 0.31, *p* = 0.06)

Examining first a cohort of 842 Parkinson’s disease patients (*Oxford Discovery* cohort [4, 6]) which had been genotyped and phenotypically characterised with 40 clinical assessments (Additional file 2: Table S1), we applied the PHENIX MPMM method to identify underlying latent continuous *phenotypic axes* that could account for the observed clinical variation. Each phenotypic axis reflected a number of co-varying observed clinical assessments. Three phenotypic axes explained more than 75% of the clinical variation, specifically Axes 1, 2 and 3 explained 39.6%, 28.7% and 6.8% of the variation respectively (Fig. 2 and Additional file 1: Fig S5). To examine whether similar phenotypic axes are obtained in different deeply phenotyped Parkinson’s disease cohorts, we derived phenotypic axes within an independent cohort of 1807 Parkinson’s disease individuals from the *Tracking UK* cohort [2] that had made similar clinical observations to the *Oxford Discovery* cohort [4, 6]. We found significant Pearson’s correlation coefficients between each cohort’s first three phenotypic axes: Axis 1 *r* = 0.92 (*p* = 3 × 10^−13^), Axis 2 *r* = 0.89 (*p* = 4 × 10^−11^), Axis 3 *r* = 0.72 (*p* = 5 × 10^−6^) (Fig. 2). Nevertheless, a major concern was that the identification of the same phenotypic axes might, at least in part, be due to the very similar structure of the clinical phenotyping between the two UK cohorts. To address this, we examined the independent US-based *PPMI* cohort [10] consisting of 439 sporadic Parkinson’s disease individuals that had been clinically phenotyped following a substantially different protocol to the UK cohorts. After deriving phenotypic axes in the *PPMI* cohort [10], we found significant similarities between the first three phenotypic axes derived for both *Oxford Discovery* [4, 6] and *PPMI* [10] cohorts: the coefficients of determination (*R^2*) between three first axes across different categories of clinical phenotypes from each cohort were: Axis1: 0.665 (*p* = 0.048), Axis 2: 0.914 (*p* = 0.003) and Axis 3: 0.754 (*p* = 0.025) (Fig. 3 and Additional file 1: Fig S6). By deriving phenotypic axis in three cohorts by using only UPDRS I, II, III and MOCA, four clinical measures systematically recorded in each cohort, we found significant similarities between the two first phenotypic axes derived in three cohorts: correlation between phenotypic axis vs clinical measure between Oxford Discovery cohorts (*x*-axis) vs others cohorts *r* = 0.92 95% [0.81–0.97]. These consistent similarities in the axes of phenotypic variation independently derived for each of three different Parkinson’s disease cohorts demonstrates the universality of these axes of phenotypic variation amongst Parkinson’s patients. Finally, by comparing PHENIX with other methods of dimensionality reduction for the UK/US cohort comparisons, specifically principal component analyses (PCA), multidimensional scaling (MDS) and independent component analysis (ICA), only the phenotypic dimensions discovered by the genetically-guided MPMM model, PHENIX, were significantly correlated between both cohorts. Hence, no other method was able to identify similar axes of phenotypic variation across UK and US Parkinson’s disease cohorts (Fig. 3).

### Each phenotypic axis represents a distinct set of clinical features

To interpret the clinical relevance of each phenotypic axis, we examined the correlation between individual clinical features and the phenotypic axes (Table 2 and Additional file 1: Fig S2 and Additional file 1: Fig S7). We observed that each phenotypic axis corresponded to a subset of clinical features, differing in both extents and directions of severity. Axis 1 represented worsening non-tremor motor phenotypes, anxiety and depression accompanied by a decline of the cognitive function (Table 2). Worsening anxiety and depression were also features of Axis 2, in addition to increasing the severity of autonomic symptoms and increasing motor dysfunction. Axis 3 was associated with general motor symptom severity including rigidity, bradykinesia and tremor of the whole body independently of non-motor features. The contribution of different phenotypes to these axes was therefore highly variable. Specific aspects of motor dysfunction were important factors in defining the majority of axes. Anxiety and depression were also relatively important features, but only for axes explaining the largest amounts of variation. Conversely, cognitive impairment was associated only with Axis 1. However, this observation must be weighted by the fact that cognitive impairment/dementia is reported at a later disease stage and thus features less in recently diagnosed cases.

**Table 2:**
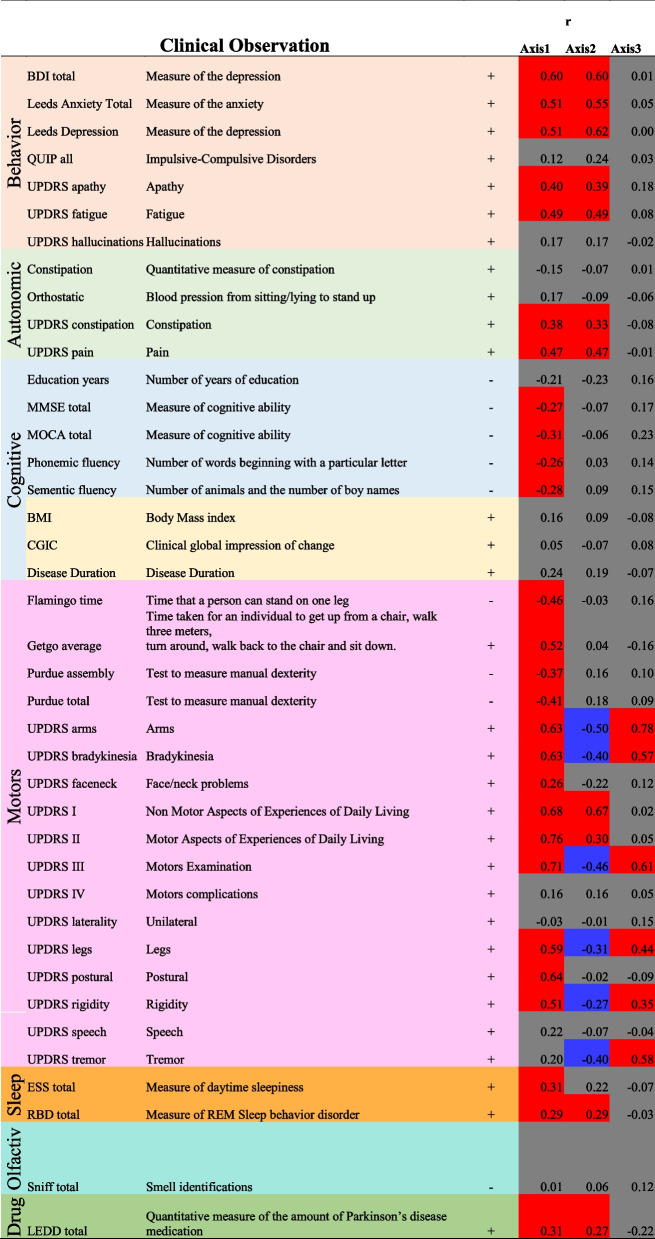
Correlation between each axis and each clinical phenotypic measure

A high score for a clinical measure indicates **more ( +)** or **less (-)** issue for the patient.

The correlation coefficient under and above |0.25| are indicated in gray or blue/red respectively

Red and blue cells indicates when a high phenotypic axis score are associated with more and less clinical issues for the patient respectively.

Although each phenotypic axis is associated with a distinct set of clinical features, they are not independent but instead strongly correlated (Additional file 1: Fig S8). We find no significant relation between the phenotypic axes and principal components of genetic ancestry (Methods) suggesting that the phenotypic axes are not biased by the population structure (Fig S9, Additional file 2 :Table S5). However, as previously reported, gender influences clinical symptoms [4] and we also observe a significant association between gender and Axis 2 (Table S5, *p* = 4.5 × 10^−5^).

To assess to what extent the phenotypic axes might be affected by the number of clinical observations, within the *Oxford Discovery* cohort [4, 6] we compared the phenotypic axes built on all clinical features with phenotypic axes generated with incomplete sets of randomly-selected clinical features. We observed a strong correlation (*r* > 0.8) between each of the two first phenotypic axes built with as few as 50% of the clinical variables and their respective original phenotypic axes, suggesting that these two axes are extremely robust in terms of the numbers of clinical variables considered (Additional file 1: Fig S9). Finally, the agreement of these phenotype axes with previously observed correlations provides further support for underlying biological themes, but their reinterpretation as robust continuous traits likely provides a more realistic approximation of how the underlying biology contributes, as opposed to a clustering-based cut-off for a phenotype. Specifically, the unimodal distribution of patients along these phenotypic axes (Additional file 1: Fig S10 and S11) suggests here that the development of continuous measures is more appropriate than clustering according to an arbitrary threshold.

### The integration of genetic relationships improves the capture of the clinical symptoms

The PHENIX MPMM approach employed here to derive phenotypic axes exploits the genetic relatedness between individuals derived from genotypic similarity to further decompose random effects into kinship effects between individuals. In its original application to imputing missing phenotypes, PHENIX outperforms other imputation approaches when the heritability (h^2^) of a phenotype increased [9]. Similarly, when randomly removing and re-imputing 10% of observed data, the quality of the imputation of Parkinson’s disease clinical assessments was in general better when considering the genetic relatedness between individuals as compared to excluding this information (Additional file 1: Fig S12), suggesting that phenotypic axes better capture Parkinson’s disease heterogeneity when including genetic information. Moreover, we found a higher agreement between the phenotypic axes derived by integrating the genetic relationship between patients of different cohorts than when the phenotypic axes were derived ignoring the genetic relationships (Additional file 1: Fig S13). Specifically, the coefficient of determination reflecting the agreement between the axes derived from *Oxford Discovery* [4, 6] and those derived from the *PPMI* [10] cohorts were from Axis 1 to 3, respectively: 0.665 (*p* = 0.048), 0.914 (*p* = 0.003) and 0.754 (*p* = 0.025) when including the genetic similarity between patients as compared to 0.604 (*p* = 0.069), 0.908 (*p* = 0.003) and 0.001 (*p* = 0.991) without. Together, these findings demonstrate that including genetic relationships between patients enhances the resulting phenotypic axes’ ability to reproducibly capture Parkinson’s disease clinical variation.

### A high Alzheimer’s genetic score increases the risk of developing a more severe Parkinson’s form

**Fig. 4:**
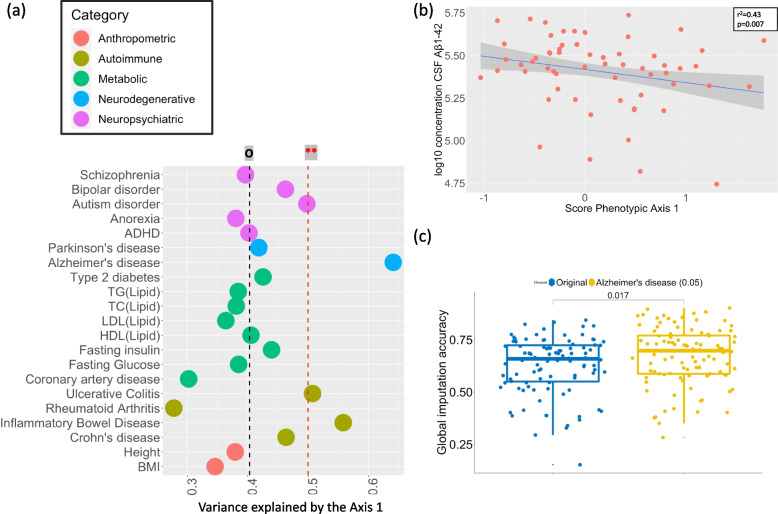
A high Alzheimer’s disease genetic risk increases the Parkinson’s disease severity risk. **a** The most influential Axis (Axis 1) is associated with the genetic risk of Alzheimer’s disease The proportion of phenotypic variation explained by the first phenotypic axis derived using these different disease risks (we considered here genome-wide association (GWA) *p*-value < 0.1) as compared to the original phenotypic axes or exceeding significantly the original phenotypic axis 1 derived using the entire genotype (black horizontal line) or random SNP set respectively (black horizontal line) within *Oxford Discovery* cohort The colour represent the category of traits: neurodegenerative, neuropsychiatric, metabolic, autoimmune and anthropometric. **b** The Axis 1 is specifically associated with a biomarker strongly associated with future conversion to dementia In the *PPMI* cohort, PD patients with higher score for the Phenotypic axis 1 (*x*-axis), have significant lower CSF level Aβ1–42 (*y*-axis), a biomarker strongly associated with future conversion to dementia. **c** The Axis 1 is associated with rapid form of Parkinson’s disease. Boxplot comparing the accuracy of PHENIX to predict the progression of different clinical phenotypes with a general relatedness genetics matrix (blue vs a genetic relatedness matrix calculated by using Alzheimer's disease's genetics variants with GWA *p* < 0.05 (yellow) in the *Oxford Discovery*, *Tracking UK* and *PPMI* cohort

To better understand the genetic risk factors influencing the phenotypic axis, we replaced the pairwise patient overall genotypic similarity matrix in the MPMM with a similarity matrix based only on regions of the genome associated with a specific complex human trait/disease. For example, replacing the overall genetic similarity with how similar people are in their genetic risk for diabetes or depression. We then rederived the phenotypic axes using the new metric of genetic similarity and compared the proportion of phenotypic variation explained by the new phenotypic axes, derived from different disease risks, to the original phenotypic axes that were derived using the entire genotype (Methods). Unexpectedly, the phenotypic axes derived using Parkinson’s disease genetic risk performed no better than the original phenotypic axes, while axes derived using the genetic risk for Alzheimer’s disease or the risk for inflammatory bowel disease, ulcerative colitis significantly outperformed, i.e. captured more patient phenotypic variation than, the original principal phenotypic Axis 1 (Fig. 4A and Additional file 1: Fig S14-15). Although UC and inflammatory bowel disease share a common genetic aetiology [23], we find no evidence that the same risk variants influence Alzheimer’s disease, suggesting that two distinct molecular aetiologies underlie phenotypic Axis 1. Specifically, we see no significant reduction in the variance explained by the axis calculated using Alzheimer’s disease genetics variants conditioned on ulcerative colitis or inflammatory bowel disease genetics variants (Additional file 1: Fig S16). The *APOE* locus is one of the major risk loci in Alzheimer’s disease, but we found no evidence that Parkinson’s disease individuals carrying one of two *APOE ε4* alleles have a significantly higher phenotypic Axis 1 score suggesting that the *APOE* locus is not a major risk locus influencing Parkinson’s disease clinical presentation (Additional file 1: Fig S17).

Our results imply that Parkinson’s disease patients with high genetic risk for Alzheimer’s disease, but excepting *APOE*, are more likely to develop a more aggressive form of Parkinson’s disease that includes dementia symptoms as indicated by Axis 1, which represents worsening non-tremor, motor phenotypes, anxiety and depression accompanied by a decline in cognitive function (Table 2 and Fig. 2). We tested this hypothesis in the *PPMI* cohort [10] and found a significant relationship between phenotypic Axis 1 and the cerebrospinal fluid (CSF) Aβ1-42 level (*r*^*2*^ = 0.43, *p* = 0.007), an Alzheimer’s-associated biomarker strongly associated with future conversion to dementia, but no correlation was observed with total Tau, phosphorylated Tau or Alpha-Synuclein levels (*p* > 0.05). Parkinson’s disease patients with a high score for phenotypic Axis 1 had a significantly lower CSF level Aβ1–42 [24, 25] (Fig. 4B).

**Fig. 5:**
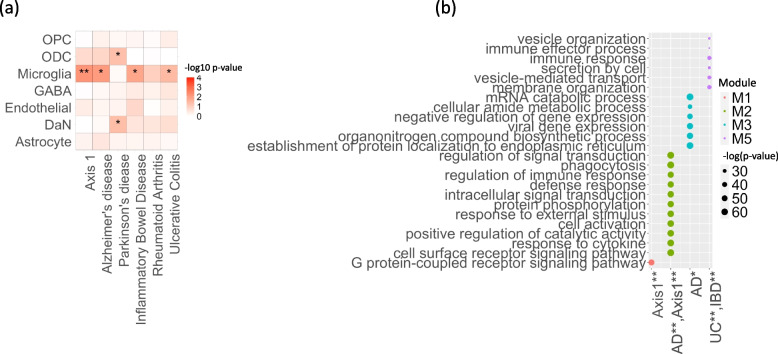
The most influential Parkinson’s disease clinical axis involves genetic pathways associated with neuroinflammation. **a** Identification of substantia nigra (SN) cell types associated with Parkinson’s disease, Alzheimer’s disease, inflammatory bowel disease and ulcerative colitis. To identify the associations between genetic risk variants of different complex traits and cell types SN, we used the MAGMA gene set analysis (one-sided positive two-sample *t*-test). The heatmap colours give different degrees of significance * and ** indicate nominally significant *p* (< 0.05) and *q* value (Bonferroni correction for the number of cell types tested) respectively. **b** Microglia-specific pathways associated with the genetic risk of the phenotypic Axis 1, Alzheimer’s disease, inflammatory bowel disease (IBD) and ulcerative colitis. Gene Ontology (GO) enrichment for substantia nigra (SN) microglia protein–protein interaction (PPI) genes modules. We tested the convergence of disease genetic risk at a functional level across SN microglia-cell specific PPI gene modules using MAGMA gene set analysis (one-sided positive two-sample *t*-test); * and ** indicate nominally significant *p*-value (< 0.05) and *q* value (Bonferroni correction for the number of PPI modules tested), respectively. The top representative GO biological process (BP) terms are shown for modules. The size of circles represents -log(*p*) for GO enrichment with Fisher’s test; colours correspond to different modules

To identify the pathways underlying the genetics of this major clinical axis, we conducted a meta-analysis for the genome-wide association study (GWAS) summary statistic of 4,211,937 variants across 3088 individuals from three cohorts (*Oxford Discovery* [4, 6], *Tracking UK * [2] and *PPMI* [10]) (Method). In line with Alzheimer’s disease risk genetics rather than Parkinson’s disease, we found an association between Phenotypic Axis 1 risk variants and both the SN and cortex microglia-specific genes, which indicates that neuro-inflammation plays a key role in the development of a more aggressive form of Parkinson’s disease (Fig. 4A). Again, following the approach of Agarwal et al. [18] we examined the intersection of genetic risk and microglia-specific functions by identifying highly connected *modules* of microglia-specific genes whose proteins interacted (Methods) and then used MAGMA to associate these functional modules with different genetic risks. Modules were then annotated with GO terms and corrected for the microglial gene expression background. The genetic risk influencing both Alzheimer’s disease and phenotypic Axis 1 converge to microglia-specific gene Module 2 (Bonferroni adjusted p-value for the number of modules Alzheimer’s disease *p* = 0.038 *–* Axis 1 *p* = 0.042), expressing proteins involved in phagocytosis and regulation of immune response (Fig. 5B).

As for Alzheimer’s disease, we also found a metabolic influence on the Parkinson’s disease phenotype. We observe that patients with a history of high cholesterol or a history of heart failure, stroke and/or heart attack score significantly higher only on phenotype Axis 1 than those without these histories (Additional file 1: Fig S18). We also observe a significant positive correlation between patient BMI and only their Phenotypic Axis 1 severity score (*r* = 0.22; *p* = 3.8e − 06; Additional file 1: Fig S19).

### A higher Alzheimer’s genetic risk increases the risk to develop a faster progressing form of Parkinson’s

In the *Oxford Discovery* [4, 6], *Tracking UK* [2], and *PPMI* [10] cohorts, we used their available repeated clinical evaluations to measure individual variation in disease progression. For each clinical phenotype, we derived a progression measure noting that the interval and span of clinical follow-ups varied between the three cohorts (Methods): on average 3.6, 3.6, 11, spanning 4.37, 4.08, and 5.58 years in the *Oxford* *Discovery* [4, 6], *Tracking UK* [2] and *PPMI* [10] cohort, respectively. The average interval between visits was 20, 19 and 6 months in the *Oxford* *Discovery* [4, 6], *Tracking UK* [2] and *PPMI* [10] cohort, respectively. We derived the longitudinal phenotypic axes by using the progression measure of each clinical phenotype. We identified a primary axis explaining 76%, 72% and 78% of the clinical progression in the *Oxford Discovery* [4, 6], *Tracking UK* [2] and *PPMI* [10] cohorts, respectively. This axis was firstly correlated with UPDRS III clinical scores for motor symptoms (*r*^*2*^_ppmi_ = 0.83 & *r*^*2*^_*Tracking*_ = 0.74, *r*^*2*^_*Discovery*_ = 0.66) and with MOCA scores for the cognitive dysfunctions (*r*^*2*^_ppmi_ = 0.69 & *r*^*2*^_*Tracking*_ = 0.65, *r*^*2*^_*Discovery*_ = 0.69). Phenotypically, this axis was significantly similar in three cohorts *(Oxford Discovery/PPMI* *r*^*2*^ = 0.68, *p* = 0.04; *Oxford Discovery/Tracking UK* *r*^*2*^ = 0.90, *p* = 3 × 10^−4^, *Tracking UK/PPMI* *r*^*2*^ = 0.70, *p* = 0.03) (Additional file 1: Fig S20). A key feature of PHENIX is the ability to impute missing data and thus potentially predict individual disease progression. Using known clinical progression and baseline clinical symptoms of 80% of patients from each cohort, we calculated the accuracy for predicting the progression measure of a clinical phenotype, given the baseline clinical features, by predicting the progression measures in the 20% of patients excluded and repeated this random exclusion and prediction 1000 times (Additional file 1: Fig S21). Accuracy for predicting the progression of cognitive dysfunction was better (*Oxford Discovery* MOCA test, *r*^*2*^ = 0.42, *Tracking UK r*^*2*^ = 0.40, *PPMI r*^*2*^ = 0.49), than predicting the progression of motor symptoms (UPDRS III *Oxford Discovery*: *r*^2^ = 0.16, *Tracking UK*: *r*^2^ = 0.29, *r*^*2*^_*Discovery*_ = 0.16). We noted that changes in olfactory function, i.e., changes in Sniffin-16 item odour identification scores in the *Oxford Discovery* cohort, showed the highest predictive accuracy (*r*^*2*^ = 0.72) when estimating deliberately left-out clinical follow-up measures. In accordance with previous studies, this suggests that hyposmic Parkinson’s disease patients exhibit a worse clinical progression as compared to normosmic patients [26]. Instead of a general genotypic relatedness matrix, using one calculated with only Alzheimer’s disease genetic risk loci significantly improved the accuracy for predicting clinical progression (*Oxford Discovery: p* = 0.017, *Tracking UK*: *p* = 0.003, PPMI: *p* = 0.04, Fig. 4D). This longitudinal phenotypic axis was significantly correlated with Axis 1 reported above that captures baseline clinical presentations (*Oxford Discovery (r*^*2*^ = 0.31, *p* < 2.2e^−16^), *Tracking UK* (*r*^*2*^ = 0.48, *p* = 4.12e^−14^), *PPMI* (*r*^*2*^ = 0.36, *p* = 4.12e^−14^)), indicating that Axis 1 is further associated with rapid progression of multiple clinical symptoms (Additional file 1: Fig S22). All these observations together indicate that genetic risk of Alzheimer’s disease could aid as a prognostic marker for Parkinson’s disease presentation and progression.

## Discussion

Understanding how and why the clinical presentations of each patient affected by the same disorder vary is a critical challenge in medicine. We demonstrate a novel approach to quantifying diverse Parkinson’s disease patient phenotypes using a continuous scale to derive phenotype axes. By applying our approach to three independent and deeply phenotyped cohorts, we demonstrate the universality of these axes of phenotypic variation amongst Parkinson’s disease patients. We also show that these axes are robustly derived when reducing the number of clinical features considered and, unlike other dimensionality reduction methods, the genetically-guided PHENIX MPMM approach is the only method tested here that is able to identify the same phenotypic axes underlying Parkinson’s disease patient variation between individuals from all three cohorts (Figs. 2 and 3).

Our approach was able to identify clinical variation that appears relevant to previously-defined categorical Parkinson’s disease subtypes. Anxiety and depression are highly correlated in Parkinson’s disease patients, both of which are correlated with Axes 1 and 2 [27]. Rigidity and bradykinesia are also linked, possibly due to shared physiology [28], and varied in the same direction along Axis 3. Lawton et al. reported five Parkinson’s disease subgroups, by using the same *Oxford Discovery* cohort [4, 6] but following a k-means clustering approach [6]. We examined the distribution of phenotypic axis scores across these five Parkinson’s disease subgroups (Additional file 1: Fig S10) and noted that the 5th subgroup of patients, characterised by severe motor, non-motor and cognitive disease, with poor psychological well-being clinical symptoms, were systematically associated with high severity score for all three of our phenotypic axes. Inversely, the first Parkinson’s disease subgroup characterised by mild motor and non-motor disease (group affected by fewer clinical symptoms) displayed a low severity score for our three phenotypic axes. Furthermore, we observed that the individuals of subgroups 4 and 5, characterised by poor psychological well-being, had high severity scores for phenotypic axis 2, the axis most associated with depression and anxiety symptoms. More recently, using both UK cohorts Lawton et al. (2018) reported four Parkinson’s disease subgroups. The subgroups with the best and worst clinical symptoms, clusters 2 and 3 associated with normal/to better and worst clinical symptoms respectively, showed corresponding variation along the phenotypic axes reported here (Additional file 1: Fig S23). These observations demonstrate some consistency between subgroups defined with k-means and our phenotypic axis severity score, but the continuous unimodal distribution of patients along the phenotypic axis does not support the existence of phenotypically distinct clusters of patients.

The phenotypic axes identified were robust in terms of the number of clinical features considered and enable the alignment of patients from different cohorts with different clinical phenotyping structures. The corollary is that PHENIX did not require the variable selection common in Parkinson’s disease clustering approaches, and it can also guide clinicians in determining which clinical assessments are essential to capture Parkinson’s disease heterogeneity. Deep phenotyping is burdensome to both patient and clinician and many of the measures exploited here are compound scores summarising aspects of functioning. Further work identifying the minimally burdensome observations that enable robust scoring of patients along these phenotypic axes would facilitate their utility and adoption across the Parkinson’s disease clinical community, bringing increased power to the discovery of influencing factors. Furthermore, the alignment of phenotypic axes across all Parkinson’s cohorts enabled a multi-cohort GWA meta-analysis of each phenotypic axis. Although no individual loci reached genome-wide significance (Additional file 1: Fig S24-S25), the universality of these axes provides a means to significantly increase power for future meta-analyses (Additional file 1: Fig S24-S25). Finally, we demonstrated here that the MPMM approach can be readily extended to include longitudinal data to determine the phenotypic axes associated with disease progression while simultaneously dealing with missing data, which is a common problem in longitudinal studies.

The phenotypic axes have multiple applications in Parkinson’s disease precision medicine. We found that Parkinson’s disease patients who carry a high genetic risk load for Alzheimer's disease are at higher risk of a more clinically aggressive Parkinson’s disease form including dementia symptoms. Parkinson’s disease patients with a high score for the Phenotypic Axis 1 had significantly lower CSF level Aβ1–42. This fits well with the previous observation that the fastest cognitive decline is with those with low CSF Aβ1-42 at diagnosis [29, 30]. This low level may correspond to PD patients affected in the same time by AD: It was reported ~ 40% of all patients with Lewy body disorders (LBD) [31] have sufficient amyloid plaque and tau tangle pathology for a concomitant Alzheimer’s disease diagnosis at autopsy and that lower Aβ1-42 levels are predictive of increasing cerebral Alzheimer’s disease and both α-synuclein pathology [32]. While CSF α-synuclein levels might increase as a result of more intense neurodegeneration in PD [33], we did not observe that Axis 1, the axis most associated with severity and progression, was significantly associated with this CSF biomarker. As obtaining CSF biomarkers is invasive, our phenotypic axis could form part of a less invasive approach to predicting PD patients most at risk for dementia.

Similar to Alzheimer’s disease onset risk but different to Parkinson’s disease onset risk [18, 34], the genetics influencing this phenotypic axis were in genomic regions enriched for microglia-expressed genes (Fig. 4A), suggesting that neuroinflammation plays a key role in the development of a more aggressive form of Parkinson’s disease. One proposition of these findings is that Parkinson’s disease progression could be significantly modified by repurposed Alzheimer’s disease-targeting therapies in some patients.

### Limitations

Our method is potentially applicable to other disorders. However, the collection of cohorts of deeply phenotyped patients for PD is unique amongst neurodegenerative disorders. The wealth of these cohorts for PD is particularly noteworthy and nothing comparable has yet been developed for other dementia disorders such as AD or frontotemporal dementia (FTD), placing PD at the cutting edge of research to identify factors underlying neurodegenerative disease/progression. This approach can be also to higher dimensional datasets such brain imaging or single cell expression dataset. However, our method makes some number of assumptions such as the phenotype being normally distributed and heritable. As a linear mixed model has been fitted to longitudinal data from all patients, the performance to predict progression may be overestimated. However, we noted the performance to predict disease progression was more accurate when using a genotypic relatedness matrix calculated with only Alzheimer’s disease genetic risk loci instead of the overall genotypic relatedness. Finally, the application of this approach requires technical expertise and the manipulation of the large genetic dataset necessitating high-performance computation*.* However, it is certainly feasible to implement an accessible and secure platform whereby available clinically-measured phenotypes and the relevant genotypic information of Parkinson’s patients could be entered and the phenotypic axes values returned, along with relevant longitudinal predictions to support clinical advice/intervention.

## Conclusions

The universal axes identified have the potential to accelerate our understanding of how Parkinson’s disease presents in individual patients, providing robust and objective quantitative traits through which patients may be appropriately compared and underlying disease-modifying mechanisms understood. This will lead ultimately to appropriately targeted therapeutic strategies delivered on an individualised basis. We believe the applications of this approach extend far beyond Parkinson’s disease.

## Supplementary Information

**Additional file 1.** Supplementary methods and figures (S1-S25).

**Additional file 2.** Supplementary tables (S1-S5).

## Data Availability

As the clinical genetic cohorts Oxford Discovery (*n* = 842) [4, 6] and Tracking UK (*n* = 1807) [2] cohorts contain potentially identifying and sensitive patient information, they cannot be publicly shared but are available upon request (https://github.com/csandorfr/Phenotypic-Axes). To access to the clinical data of the Oxford Discovery cohort, researchers must to complete the following form https://www.dpag.ox.ac.uk/files/research/opdc-biosample-and-clinical-data-application-form send it to Prof Richard Wade-Martins (email richard.wade-martins@dpag.ox.ac.uk) and Prof Michele Hu (email michele.hu@ndcn.ox.ac.uk). To access to the clinical data of the Tracking UK cohort, researchers must contact Dr Donal Grosset (donaldgrosset@gmail.com). PPMI data are available to the research community on the PPMI website: www.ppmi-info.org PHENIX code used here is available at the following link: https://mathgen.stats.ox.ac.uk/genetics_software/phenix/phenix.html. An example illustrating how to derive phenotypic axes with PHENIX can be found here https://github.com/csandorfr/Workshop-PhenoAxis-Lux-Feb2019. The code to derive the measures of disease progression and severity lmm can be found here: https://github.com/aschalkamp/PhenotypicAxes_DiseaseProgression. The code associated with the different figures of this manuscript can be found here: https://github.com/csandorfr/Phenotypic-Axes. The cell association analyses have been performed with the same approach and dataset described by Agarwal et al. (2020) [18]: The processed 10 × 3′ Chromium single-nuclei RNAseq UMI-barcode matrices for each sample are available from the Gene Expression Omnibus under the accession code GSE140231 [18] (https://www.ncbi.nlm.nih.gov/geo/query/acc.cgi?acc=GSE140231). An R Markdown document, including a version with R code to generate these gene sets and perform cell-type association, can be found here: https://github.com/csandorfr/SN_Atlas.

## Abbreviations

BMI: Body mass index
BP: Biological processes
CIs: Confidence interval
DA: Dopamine
GBA: Glucocerebrosidase
GO: Gene Ontology
GWAS: Genome-wide association study
h2: Heritability
ICA: Independent component analysis
LD: Linkage disequilibrium
MDS: Multidimensional scaling
MPMM: Multiple phenotype mixed model
MRC: Medical Research Council
NIHR: National Institute for Health Research
OPDC: Oxford Parkinson’s Disease Centre
PCA: Principle component analyses
PHENIX: PHENotype Imputation eXpediated
PPI: Protein-protein interaction
PPMI: Parkinson’s Progression Markers Initiative
Q-Q: Quantile-quantile
SN: Substantia nigra
