# Supplementary material for: Universal clinical Parkinson’s disease axes identify a major influence of neuroinflammation
Source: Genome Med. 2022 Nov 16;14:129. doi: 10.1186/s13073-022-01132-9 (PMC9670420; doi:10.1186/s13073-022-01132-9)
Supplement: Supplementary file 1 — Additional file 1. Supplementary methods and figures (S1-S25). [file 13073_2022_1132_MOESM1_ESM.docx]

Supplemental Material:

Universal clinical Parkinson’s disease axes

identify a major influence of neuroinflammation.

Materials & Methods

**Genotype: quality control**

Quality control was carried out independently using PLINK v1.9 [1]. Variants were excluded if minor allele frequency (MAF) was less than 0.01, Hardy-Weinberg Equilibrium p-value was less than 1x10^-5^ and/or missing data rate was above 5%. Individuals were excluded if genotypic and phenotypic sex was discordant, missing data was greater than 2%, and/or heterozygosity rate was greater than two standard deviations from the mean. Principle component analysis (PCA), using EIGENSTRAT [2], included additional individuals of Central European descent from the International HapMap Project (release 23) [3]. Samples of non-European ancestry were then identified. Individuals were excluded if their score was greater than 6 standard deviations from the mean for any of the first 10 principal components.

**Genotype Imputation**

Imputation of unobserved and missing variants was performed separately for each cohort. A reference panel containing comprehensive SNP data was used to identify extended patterns of Linkage Disequilibrium (LD) and co-inherited alleles. The Michigan Imputation Server was used to phase and impute data for both cohorts separately, using Eagle and Minimac3 respectively [4, 5]. The 1000 Genomes project (phase 3, release 5) contained data for over 80 million variants in 503 individuals of European descent and provided the reference panel [6]. For each SNP an r^2^ value was produced reflecting the accuracy of imputation at that locus. Variants were filtered for r^2^ higher than 0.3 to ensure that only well-imputed SNPs were used in further analysis. Only variants with a minor allele frequency above 0.01 in both cohorts were used in association testing, as rare alleles were challenging to impute reliably and could have been prone to false positive associations in small samples.

**Imputation for Apolipoprotein E ε Alleles**

We defined different APOE alleles carried by each individual with genotype or imputed genotype for two following SNP rs429358 and rs7412.

**Using PHENIX**

We used PHENIX (version 1.0) that can be downloaded here:

<https://mathgen.stats.ox.ac.uk/genetics_software/phenix/phenix.html>

We ran PHENIX with the following command: Phenix (P, G, seed=8473, quantnorm=TRUE, scale=TRUE, trim=FALSE). The options quantnorm, scale and trim are important because they determine how PHENIX treats the data before generating the components (see documentation of PHENIX). P and G represents the Phenotypic Matrix and the Kinship matrix respectively (see section Kinship matrix and Phenotypic Matrix).

**Kinship matrix**

The kinship matrix G, used by PHENIX to identify related individuals, was created using the GEMMA software (version 0.95) [7-9] from genotype file under plink binary format. We pruned out SNPs in LD by using the following plink commands:

(1) ./plink - - bfile *plink binary files* - - indep-pairwise 50 5 0.2 - - out *output filename 1*

(2) ./plink - - bfile *plink binary files* - - exclude *output filename 1* - - make-bed - - out *output filename 2*

We then ran GEMMA with the following command:

./gemma -bfile *plink binary files of Parkinson’s disease cases with pruned SNPs* -gk 2 -o *output filename*

*Phenotype Matrix*

We generated phenotypic matrix P by recoding all categorical variables into numeric variables., e.g., the constip_cat variable was given as "<1 or laxative use", “1", “2", “>2", was recoded to 0,1,2,3 respectively. If any categorical variables were inferred from continuous measures, e.g., mild/moderate/severe cognitive impairment inferred from Beck’s depression inventory or hyposmia inferred from Sniffin, we chose to only utilise the continuous measure. We used the unadjusted score for the education years and included it as an additional phenotype. To derive the phenotypic axes independently of the genetic relationship between individuals, we replaced the Kinship matrix by the identify matrix.

**Phenotypic axis identity**

MPMM is invariant to joint rotations of the latent traits, S, and their effects, β. In particular, the three axes of clinical Parkinson’s disease variation identified here could be changed and still fit MPMM just as well. PHENIX solves this non-identifiability implicitly by initializing S and β with PCA (with missing values replaced by trait-wise means). As a result, the alternating minimisation strategy in PHENIX approximately retains the structure of its initialization, meaning (i) phenotypic axes can approximately be considered refinements of top PCs and (ii) are robust in practice despite their formal non-identifiability. We verified this assumption by downsampling the *Oxford Discovery* cohort to 90% of its original size, recalculating phenotypic axis for this sub-sample, and assessing consistency with the original phenix components. We repeated this process 10,000 times and found that the correlations were always large, ranging from 0.96 (sd=0.004), 0.97 (sd =0.003) and 0.95 (sd=0.007) for Axis 1, 2, and 3 respectively.

*Phenotypic axis in the Tracking UK cohort*

Leon Hubbard generated phenotypic axes with the same workflow described above.

**Fraction of the clinical variation explained by phenotypic axis**

To estimate the fraction of clinical variation explained by each Axis, we used the factor loading matrix, out$beta in PHENIX output with following R command:

all_pves <- matrix( NA, M, P )

for( m in 1:M ) {

for( p in 1:P) {

var_exp <- var(out$S[,m]*out$beta[m,p])

var_tot<-var(out$imp[,p])

all_pves[m,p] <- var_exp / var_tot

}

}

pves <- rowSums( all_pves )

*Correlation of individual clinically measured Parkinson’s disease phenotypes with the phenotypic axes*

We computed the Pearson's correlation coefficient and its significance for individual observed phenotypes and each phenotypic axis. Here we used the input matrix phenotype P matrix provided to PHENIX for individual observed phenotypes, which included missing data.

*Comparison of MPMM and other reduction dimensionality methods*

We compared the ability of different dimensionality reduction methods including MPMM to produce the same phenotypic axes for Parkinson’s disease patient variation between the *Oxford Discovery (UK)* and *PPMI* (US) Parkinson’s disease patients. For each cohort, we derived the continuous variable underlying multiple clinical observations by using different dimensionality reduction methods including:

(1) phenotypic axis based on a multiple phenotype mixed model (MPMM, see PHENIX paragraph)

(2) independent component analysis (ICA).

We used the function *fastICA* of the fastICA R package. We extracted 3 components.

(3) Multidimensional scaling method (MDS).

We computed the Euclidean distance between individuals from phenotypic matrix and ran the R function *cmdscale* on the distance matrix by considering 3 dimensions

(4) Principal component analysis (PCA)

We performed a PCA by using the R function *prcomp* after scaling the variables. For each analysis, we used the input matrix phenotype P matrix provided to PHENIX, but replaced the missing data for each phenotype with the mean of the observations.

We computed the Pearson's correlation coefficient of individual observed phenotypes with each phenotypic axis. As the clinical structure of the *PPMI* and *Oxford* *Discovery* cohort was different, we calculated the correlation at the level of Parkinson’s disease symptom categories: Anxiety & Depression, Autonomic, Cognitive, Motors, Olfactive and Sleep. We then compared the correlation coefficients generated from both cohorts to evaluate the ability of an approach to produce the same phenotypic axes of Parkinson’s disease patient variation in the two independent cohorts. For each method and each dimension, we then regressed the normalized coefficient derived from the *PPMI* cohort as well as the *Oxford Discovery* cohorts using the number total clinical features as the weighting factor (**Fig.2**) .

**Quantitative trait GWAS**

A quantitative trait Genome Wide Association Study (GWAS) was carried out using PLINK [1]. Linear regression was performed on scores for each phenotype axis to identify SNPs affecting its severity. Effects were analysed under an additive model that conditioned on age, sex and the first two principal components to account for any underlying population substructure. We ran the following plink command:

plink --bed *plink bed file* --bim *plink bim file* --covar *covariates.txt* --fam *plink fam file * --hide-covar --linear --maf 0.05 --missing-code NA --out *output filename 1*

Only SNPs with minor allele frequency greater than 0.01 were investigated, as this cohort was relatively small and consequently underpowered to detect true associations of rare variants.

**GWAS Meta-analysis**

We then performed a GWAS meta-analysis by using the summary statistics of three individual quantitative trait GWAS with METAL [10]. We used the following METAL command: metal *metal parameters file*

The *metal parameters file* had the following format:

MARKER SNP

ALLELE A1 A2

PVALUE P

EFFECT BETA

WEIGHT NMISS

PROCESS *Metal file association OPDC cohort*

PROCESS *Metal file association Tracking cohort*

PROCESS *Metal file association PPMI*

ANALYZE

As the same severity axis could be inverse between cohorts, we examined the correlation sign between a severity axis and different clinical assessments. We modified the sign of the Beta value (the summary statistic of the linear regression) to conserve the same correlation sign between clinical assessments and phenotypic levels derived from different cohorts.

Figures


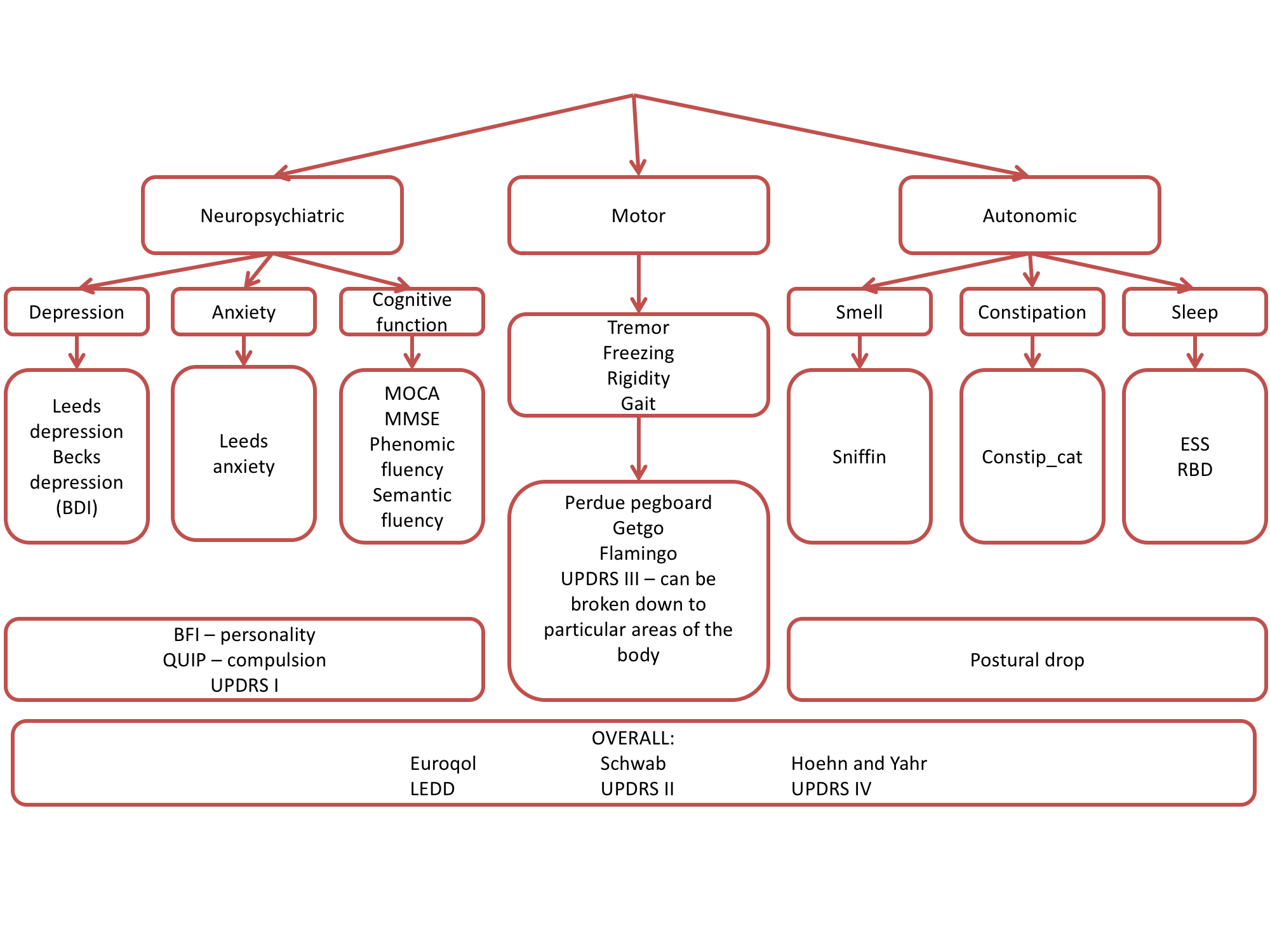


**Fig S1: Clinical features collected in the *Oxford Discovery* cohort.**

The *Oxford Discovery* cohort is a well-characterised cohort of people with Parkinson's disease. Phenotype and genotype data from 842 Parkinson’s disease cases from the *Oxford* *Discovery* cohort were used in this analysis. Phenotype data were collected for over 50 attributes listed in the **Additional file2:Table S1**, encompassing autonomic, neurological and motor system and listed in detail in the flowchart.

**
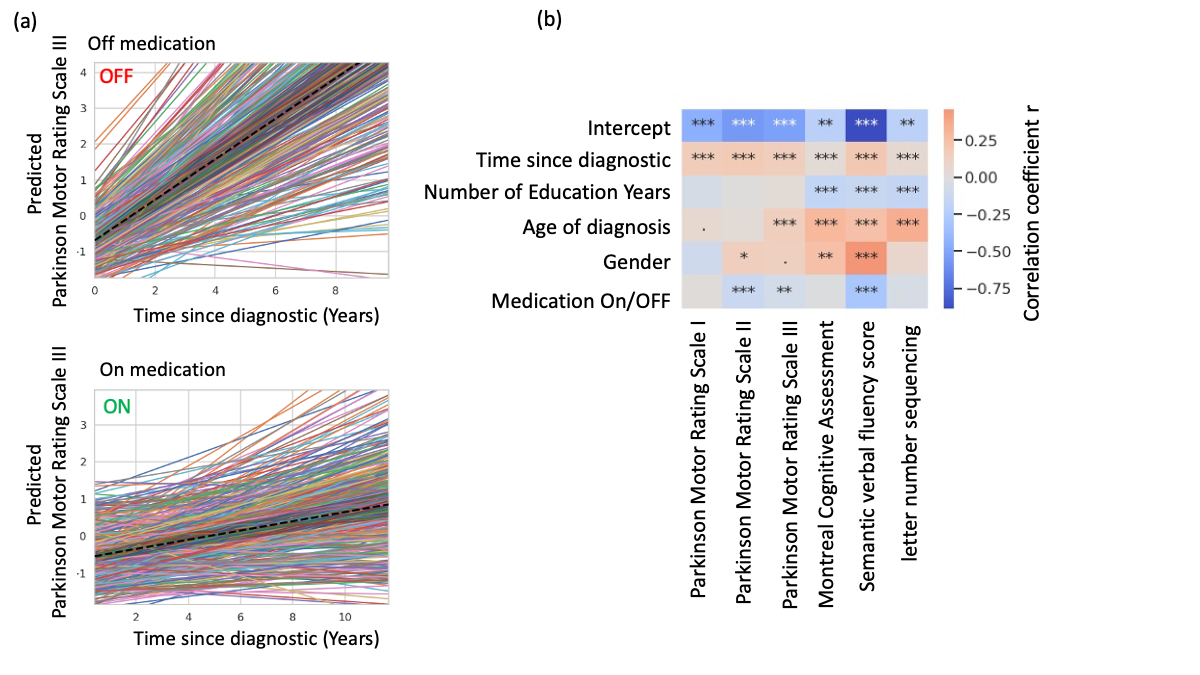
**

**Fig S2: Major factors influencing the progression of different Parkinson’s disease (PD) clinical phenotype**

We used a linear mixed model to derive the progression of different clinical phenotypes in the *PPMI* cohort. (a) Taking (bottom) or not taking (top) medications influence the progression of Parkinson Motor Rating Scale (UPDRS) III clinical score, a major clinical phenotype in PD. Patients not taking medication have faster progression in UPDRS-III clinical score than those that do. Each line represent the clinical progression of an individual for UPDRS III predicted with a lmm.
(b) Other factors (y-axis: time of diagnosis, education years diagnosis age, gender and taking or not taking medication) influencing the progression of different PD clinical phenotypes (x-axis: UPDRS I,II,III, Montreal Cognitive Assessment, semantic fluency and letter number sequencing. The color of the heatmap represents the correlation coefficient r^2^, while the symbol within each cell represents the p-value (p) associated with r^2^: “***”, “**”, “*”, “.”, correspond to p < 0.001, p< 0.01, p < 0.05, p < 0.1 respectively.


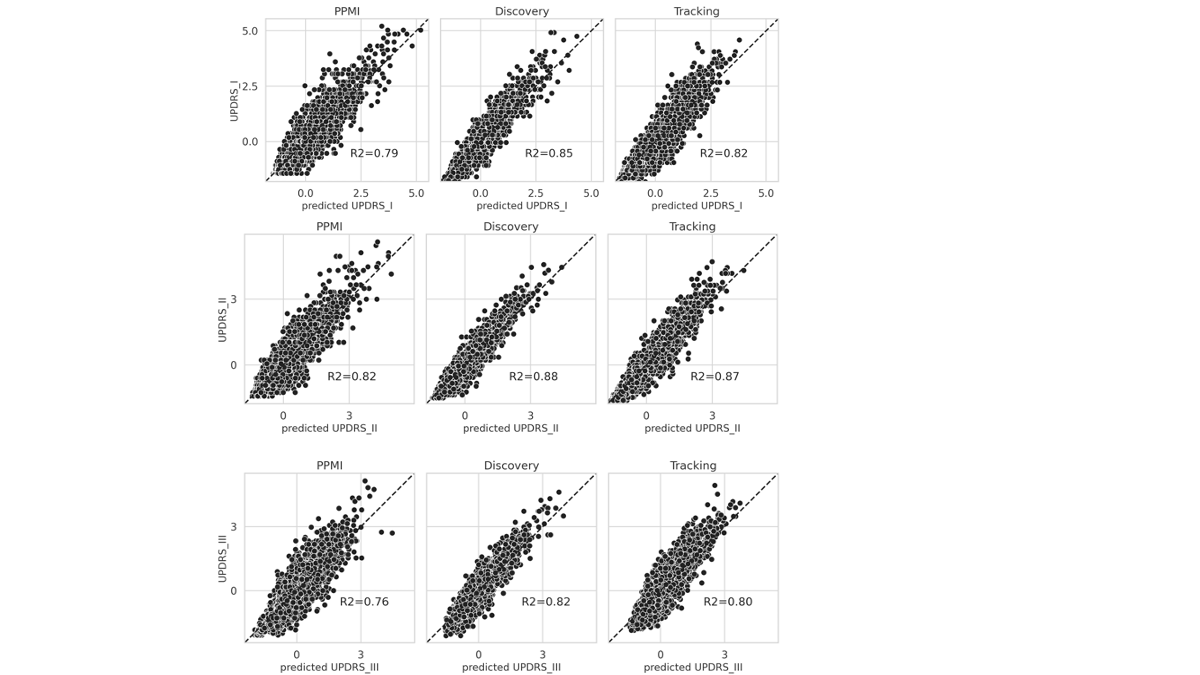


**Fig S3: Diagnostic plot of the linear mixed effect models (LMM) used to clinical score of severity at diagnosis & progression for UPDRS I, II and III** The goodness of LMM was estimated as the R^2 once with random effects


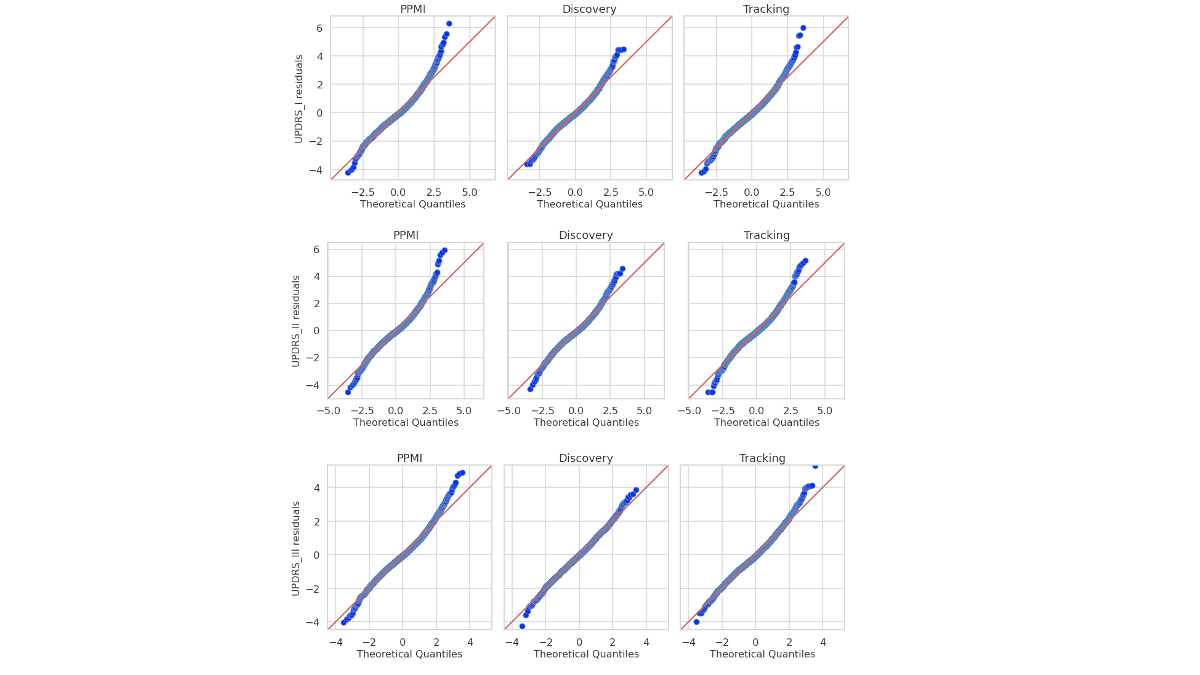


**Fig S4: Normal Q-Q. Distribution of Linear Mixed Model residuals for for UPDRS I, II and III**


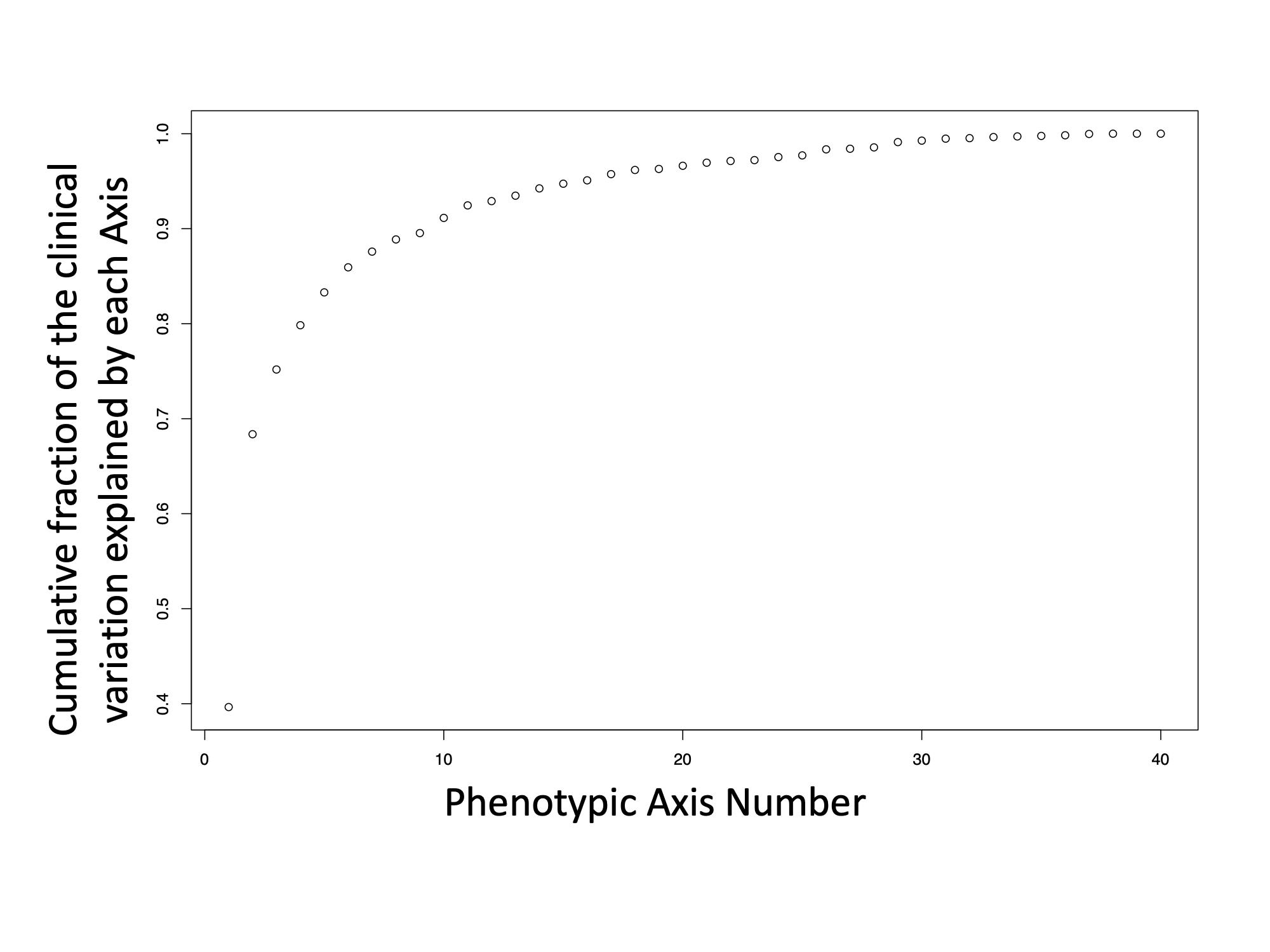


**Fig S5: Cumulative fraction of the clinical variation explained by each axis in the *Oxford Discovery* cohort** (**Methods**).

In total, the first three axes explained 75% of the clinical variation.


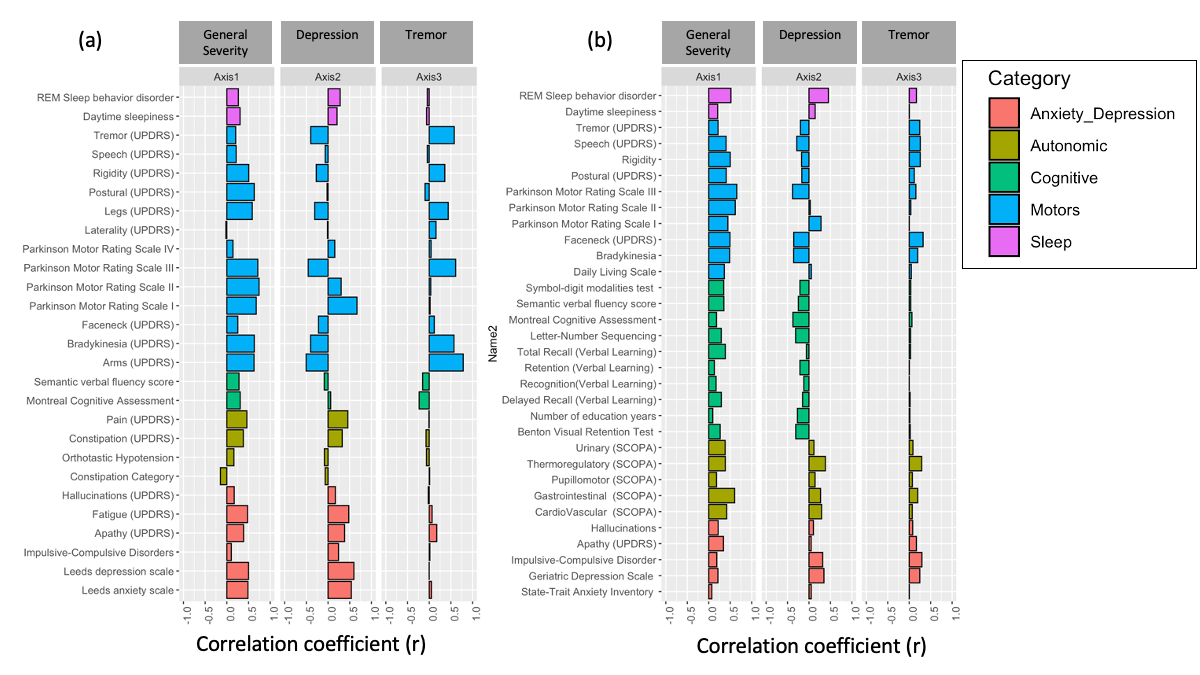


**Fig S6: Phenotypic axes identified in a deeply but very differently phenotyped US Parkinson’s disease cohort align with the same categories of clinical symptoms as previous UK-cohort derived phenotypic axes.**

We derived the phenotypic axes within a third very differently phenotyped independent cohort: US-based *PPMI* cohort including 439 sporadic Parkinson’s disease individuals with a different clinical phenotyping structure than the 2 UK Parkinson’s disease cohorts (*Oxford Discovery* & *Tracking UK*). The bar plots **(a)** and **(b)** show the correlation (correlation coefficient **r**, x-axis) between three first phenotypic axis and clinical observations (y-axis) in the *Discovery* and *PPMI* cohort, respectively. We represented six major categories of Parkinson’s disease symptoms by the colour of the bar plots. These categories include anxiety and depression, the autonomic system, cognitive functions, the motor system, the olfactory system and sleep disorders. As specific clinical measures differed between these cohorts, we compared how correlated each axis was across each specific category of Parkinson’s clinical observations. For this, we recorded the mean of the correlation coefficients of an axis across clinical observations associated with a category of Parkinson’s disease symptoms and then compared these correlations coefficient by symptom category in both cohorts. The correlation between the first three axes with a different category of clinical manifestation from each cohort was r=0.85 (p=1x10^-7^).


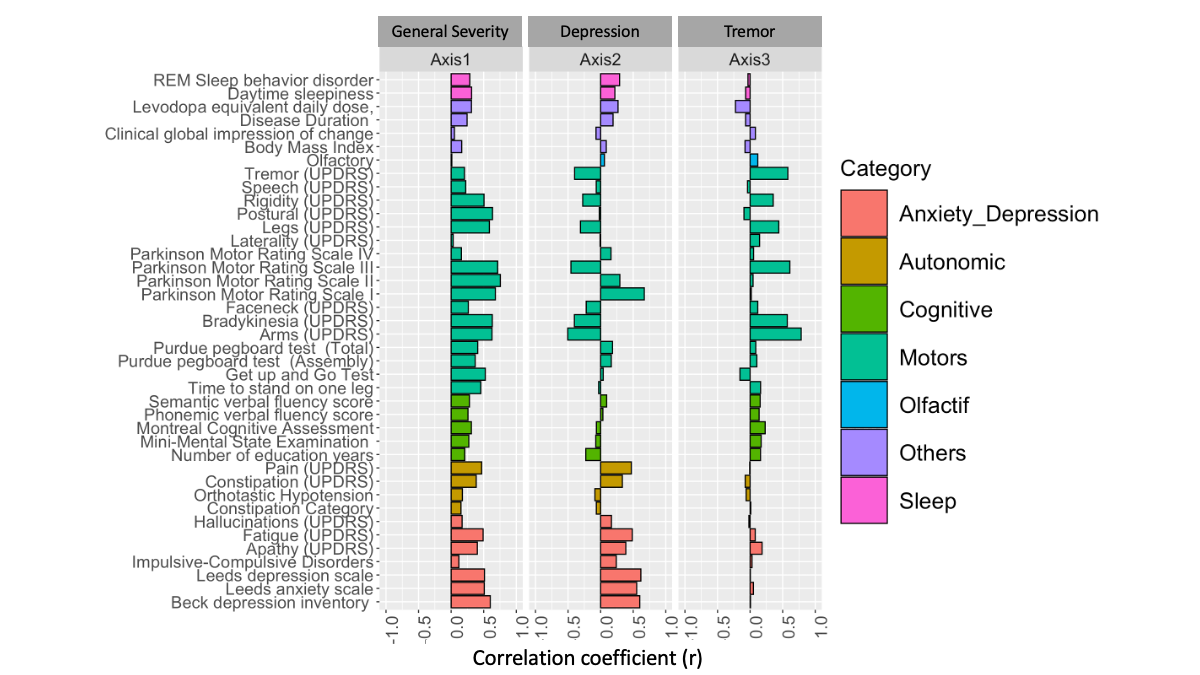


**Fig S7 Each phenotypic axis represents a distinct set of clinical features.**

For the first three phenotypic axes (each panel), we found the correlation (Pearson’s correlation coefficient **r**, x-axis) between each axis and each clinical phenotypic measure (y-axis). The colour gradient of bars is proportional to the r coefficient. The axes can be broadly summarized as: Axis 1 - Worsening anxiety, depression and non-tremor motor and cognitive decline, Axis 2 - worsening anxiety, depression and autonomic symptoms but minimal motor dysfunction, Axis 3 - Rigidity, bradykinesia and tremor in limbs, face and neck.

**
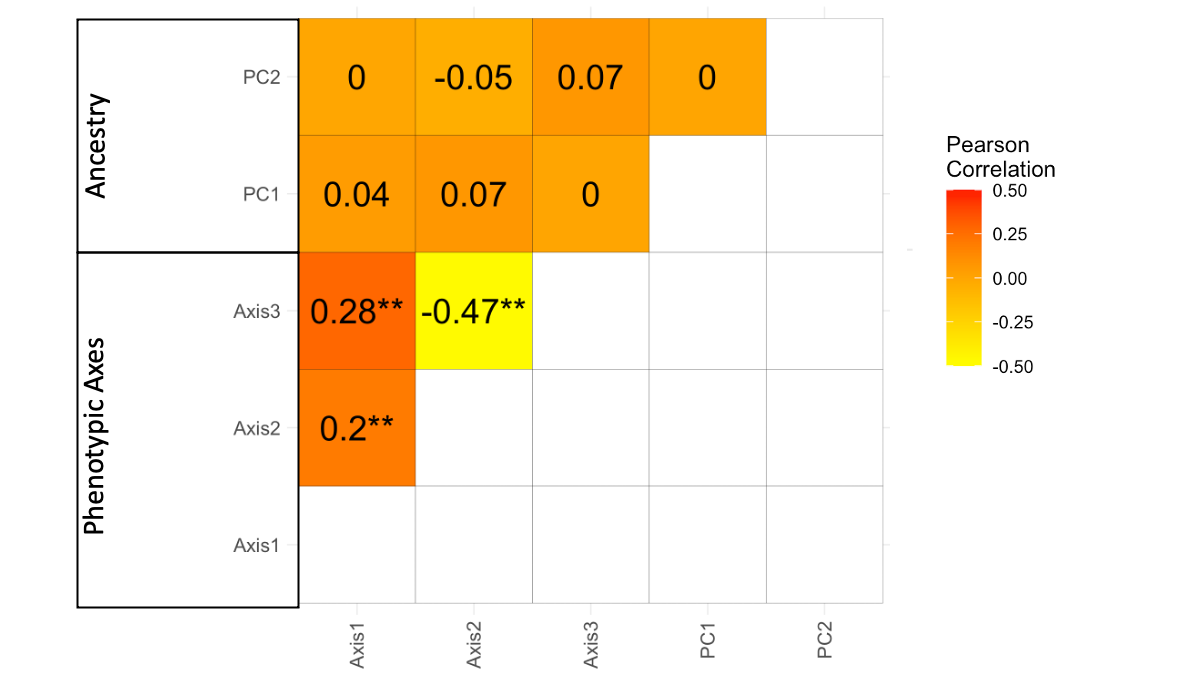
**

**Fig S8: Correlation between the phenotypic axes and the axes of the genetic variation.**

We generated the axes of genetic variation inferred by PCA (Methods)  *for* Parkinson’s disease individuals from three cohorts (*Oxford* *Discovery, Tracking UK and PPMI*) and We then examined the correlation (Person’s correlation coefficient) between the three phenotypic axes and the two first axes of genetic variation. The heatmap represents the correlation matrix. The * and ** symbols indicate, the correlations that are significant with a nominal p-value < 5% and q-value (Bonferroni correction) < 5% respectively**
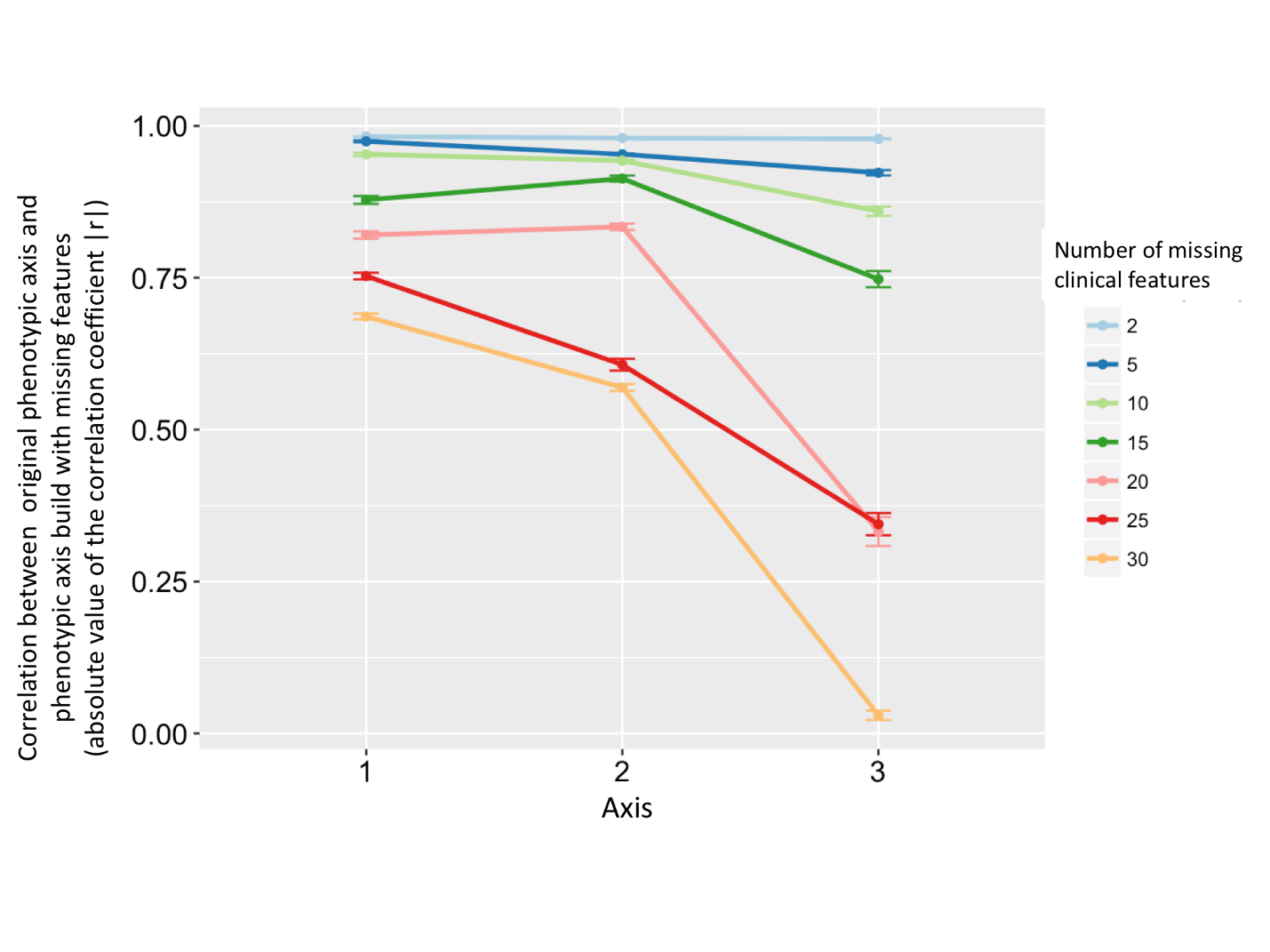
 Fig S9:**  **Comparison between the original phenotypic axes identified using all data and phenotypic axes regenerated with a proportion of randomly missing features.**

We computed the correlation between the phenotypic axes generated with all clinical data with phenotypic axes built by randomly removing clinical features. The x-axis and y-axis are the axis number and the absolute value of correlation coefficient respectively.

**
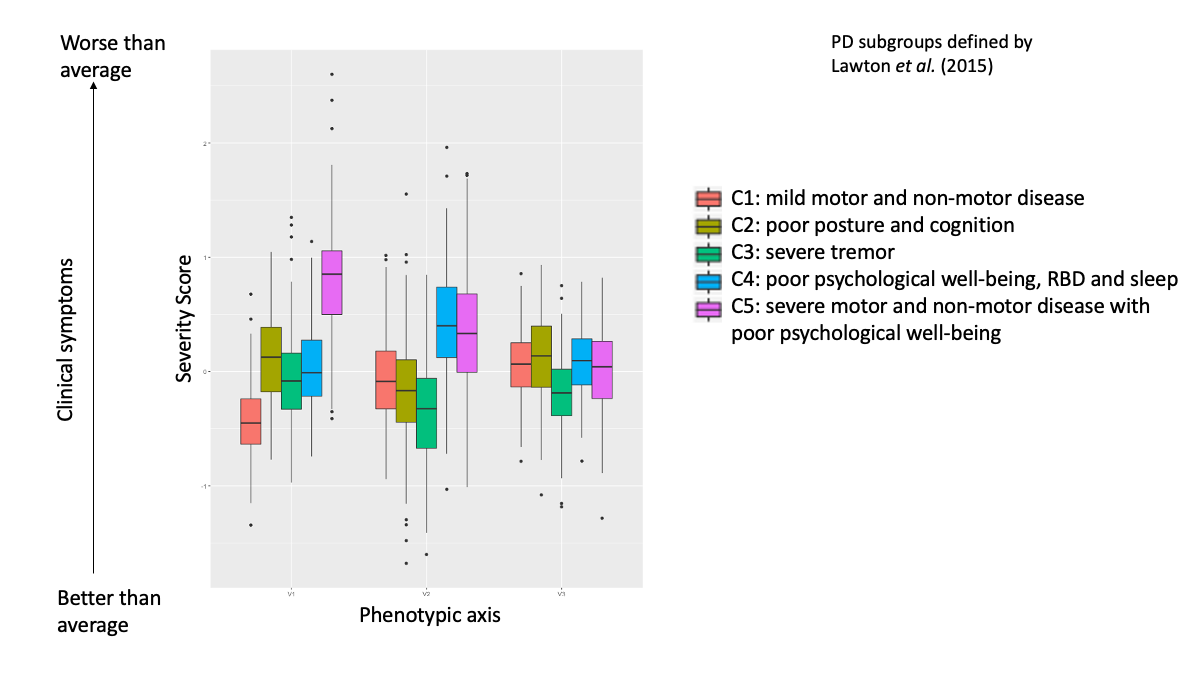
**

**Fig S10: Comparison between the phenotypic axes’ severity score and the Parkinson’s disease subgroups defined previously using a K-means clustering approach.**

We examined the distribution of phenotypic axes scores (y-axis) (x-axis represents phenotypic axis number from one to three) according to the Parkinson’s disease subgroup (colour of bars) defined previously with a K-means clustering approach [11] for 684 *Oxford* *Discovery* Parkinson’s disease individuals. A positive and negative value on the y-axis represents clinical symptoms worst and better than the average respectively.


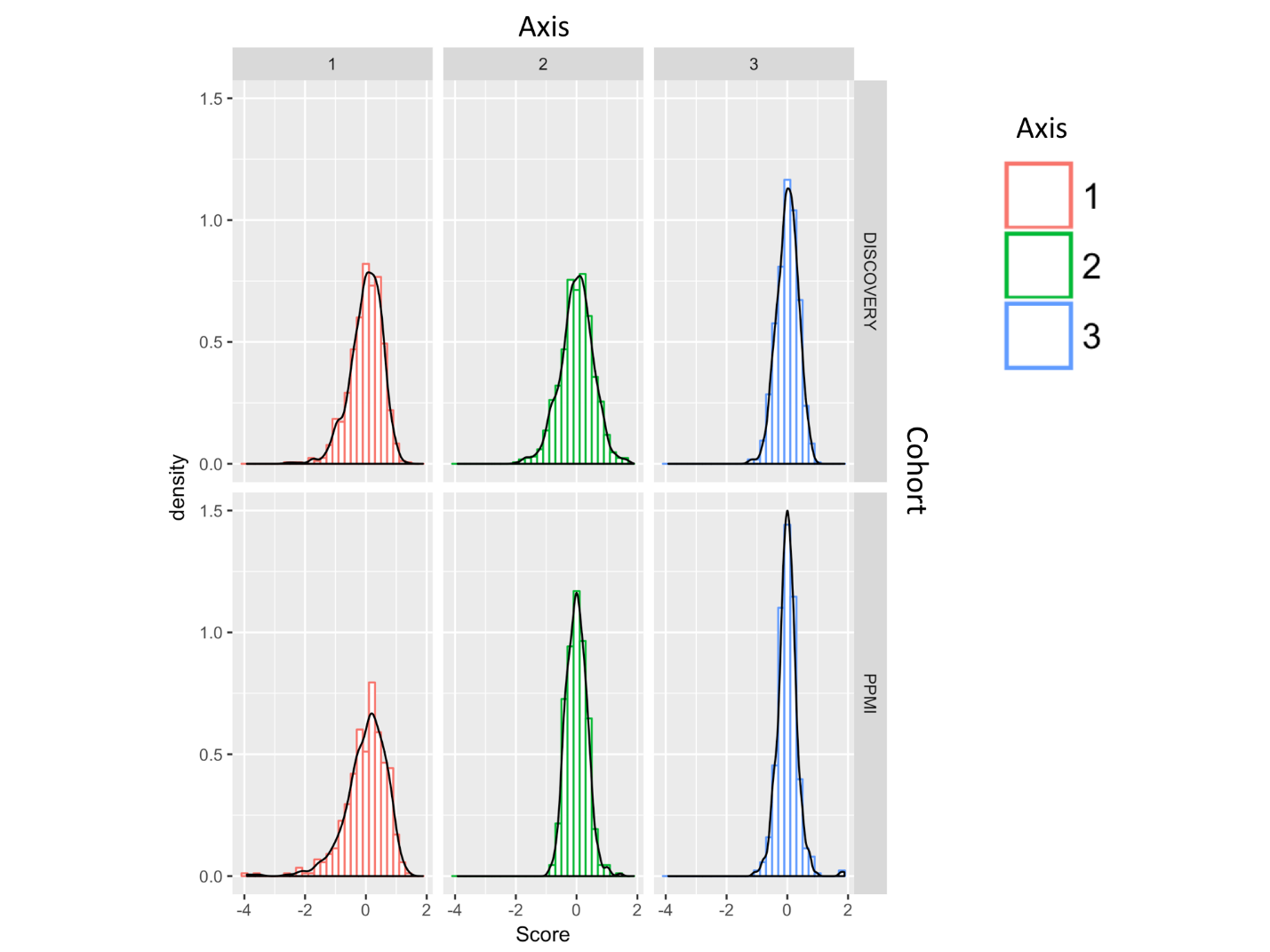


**Fig S11: Distribution of phenotypic axes in two cohorts: *Oxford Discovery* and *PPMI* cohorts.**


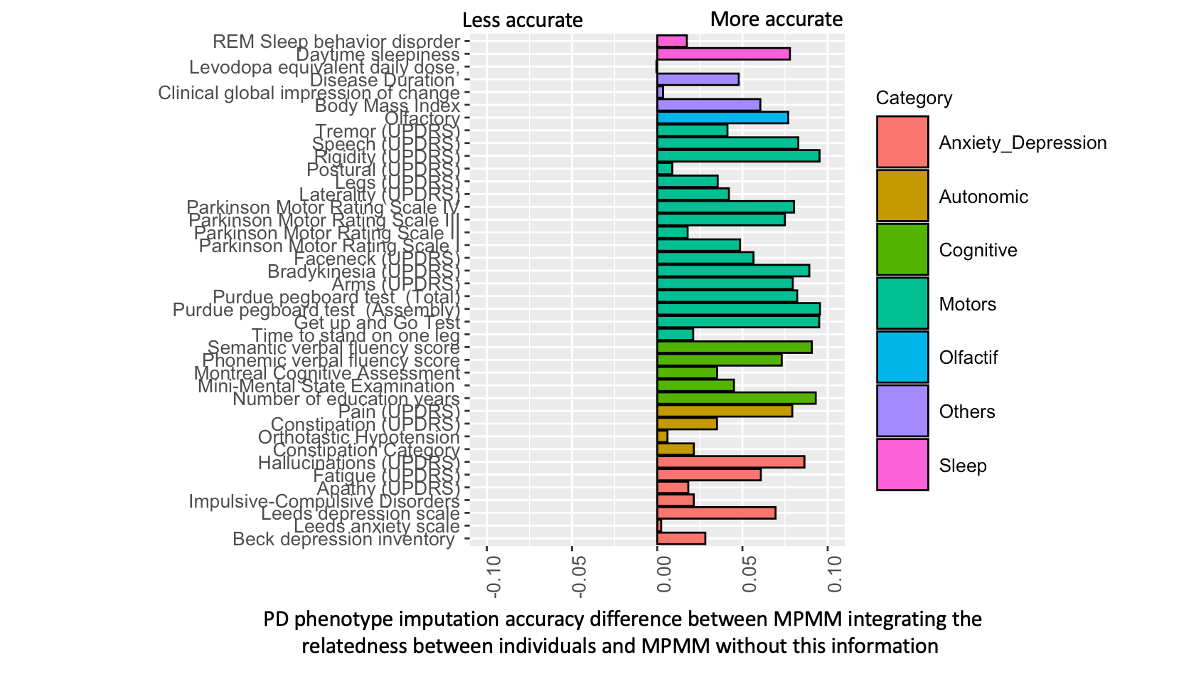


**Fig S12: Comparison of the imputation accuracy for different clinical observations with/without the integration of genetic relationship to impute missing data.**

5% of data have been randomly removed for different clinical features and re-imputed with PHENIX by using either the original kinship matrix or by replacing the kinship matrix by the identity matrix. We then calculated the correlations between the true masked observations and the imputed data for each clinical phenotype. The bar plot shows the difference between the imputation accuracy with or without the integration of genetic relationship to derive the phenotypic axes for the different clinical features.

**
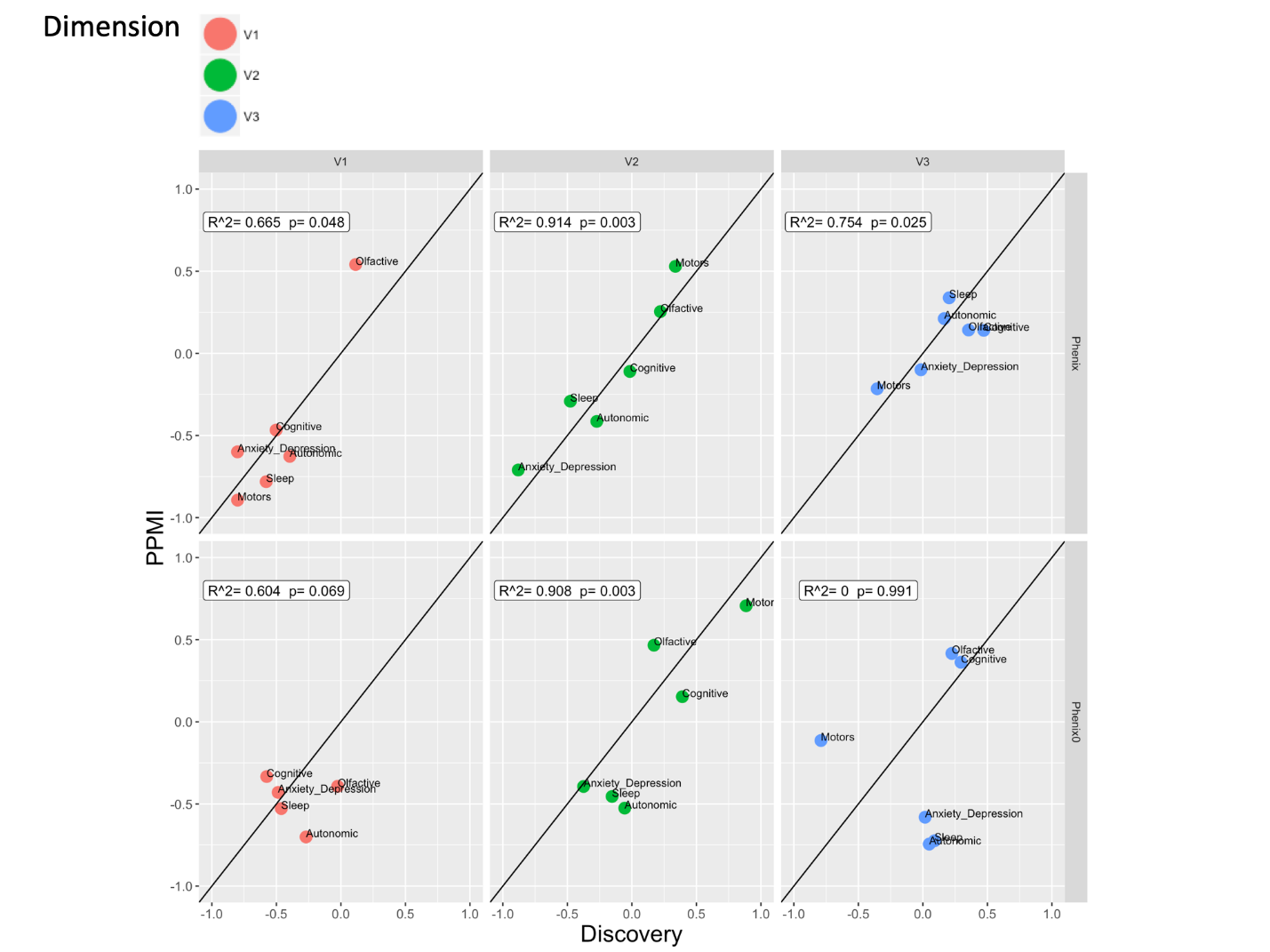
**

**Fig S13: The inclusions of genetic relationships between patients increases the phenotypic axes’ ability to capture the same Parkinson’s disease clinical variation in two different cohorts.**

The phenotypic axes were derived in two independent cohorts by integrating (top panel) or not integrating (bottom panel) the genetic relationship between patients.

We then examined each derived variable for specific categories of Parkinson symptoms (Anxiety & Depression, Autonomic, Cognitive, Motors, Olfactive and Sleep) were correlated between the two cohorts (Method). The x-axis and y-axis represent the correlation coefficient between each continuous variable representing specific symptom categories in *Oxford Discovery* and *PPMI* cohort respectively. Each columns and colour represent the dimension level of each underlying variable. All points on the diagonal would represent a perfect phenotypic alignment of two cohorts. We examined the relationship between correlation derived from both cohorts by performing a linear regression: R^2^ and p correspond to the coefficient of determination and the p-value respectively. We found a higher agreement between phenotypic axis derived by integrating genetic relationship between patients of different cohorts than the phenotypic axis estimated without the genetic relationship between individuals: The coefficient of determination R^2^ were 0.86 (p-value = 2e-08) and 0.58 (p-value = 1e-04) respectively.


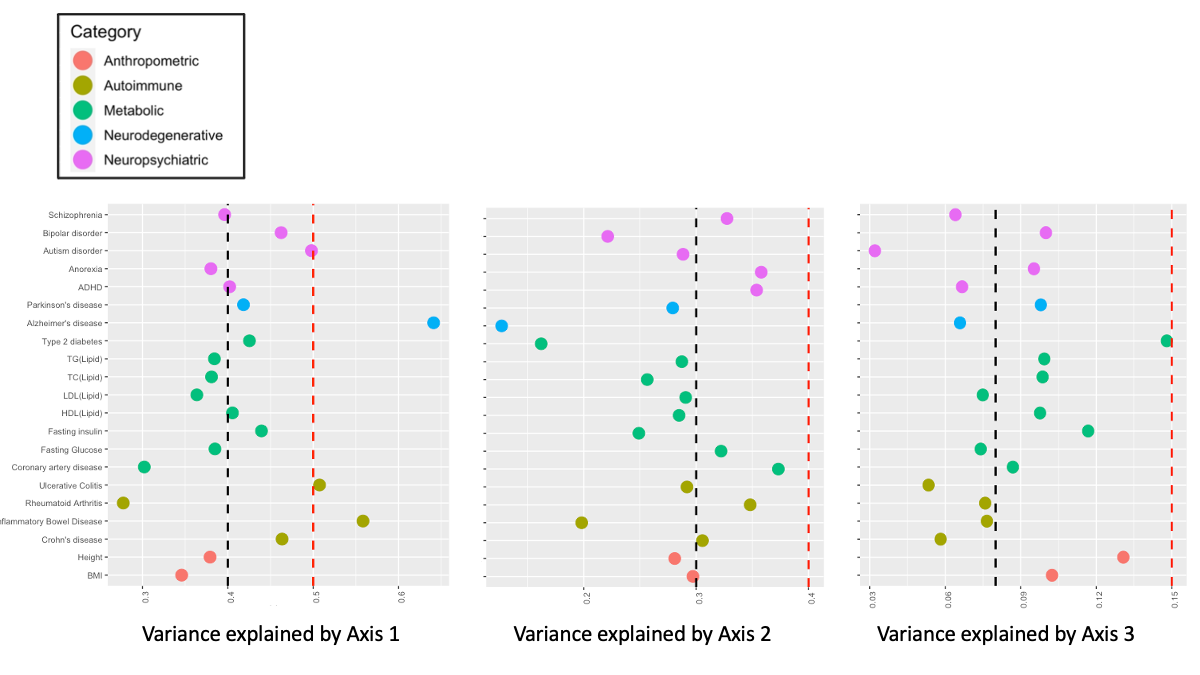


**Fig S14:** The proportion of phenotypic variation explained by the three first phenotypic axis derived using these different disease risk (we considered here genome-wide association (GWA) p-value < 0.1) as compared to the original phenotypic axes or exceeding significantly the original phenotypic axis 1,2,3 derived using the entire genotype (black horizontal line) or random SNP set respectively(black horizontal line) within Oxford Discovery cohort The color represent the category of traits: Neurodegenerative, Neuropsychiatric, Metabolic, Autoimmune and Anthropometric.

**
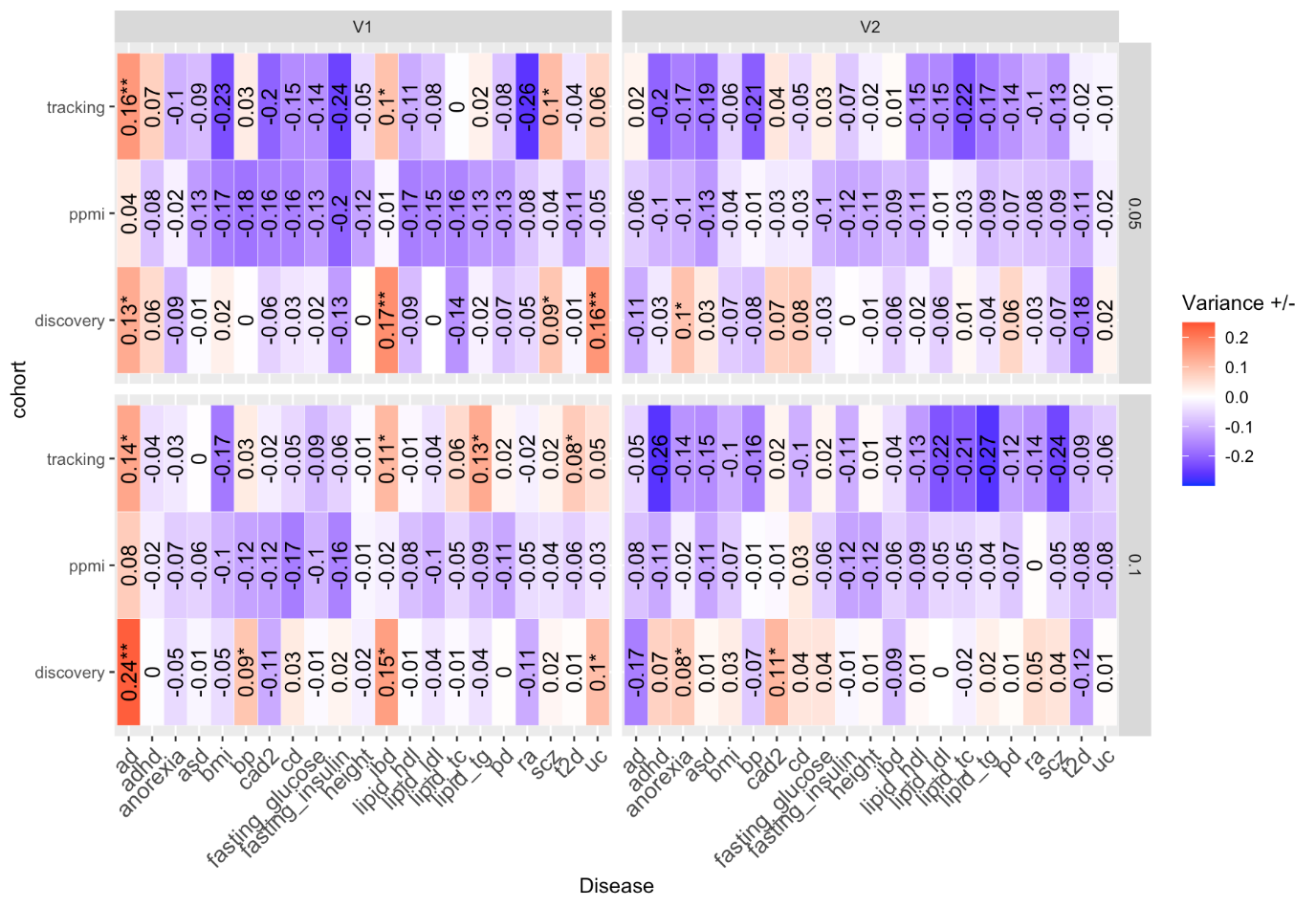
**

**Fig S15: Alzheimer’s risk-guided phenotypic axis significantly outperforms the original phenotypic axis.** These heatmaps represent the excess (red) or deficit (blue) of the phenotypic variance explained by the two first phenotypic axes (left=V1 and right=V2), in three cohorts (row 1= *Tracking UK*, row 2=*PPMI*, row 3=*Oxford Discovery* ) compared to the original phenotypic axes. Different traits from left to the right are: Alzheimer's disease (ad), Attention deficit hyperactivity disorder (adhd), Anorexia, Autism disorder (asd) , Body Mass Index (bmi), Bipolar disorder (bp), Coronary artery disease (cad2), Crohn’s disease (cd), Fasting Glucose, Fasting insulin, Height, Inflammatory Bowel Disease (ibd), Autoimmune, plasma level of high-density lipoproteins concentration (lipid_hdl), low-density lipoproteins (lipid_ldl), Total cholesterol (tc_lipid), Triglycerides (tg_lipid), Parkinson's disease (pd), Rheumatoid Arthritis (ra), Schizophrenia (scz), Type 2 diabetes (t2d) and Ulcerative Colitis (uc).

**
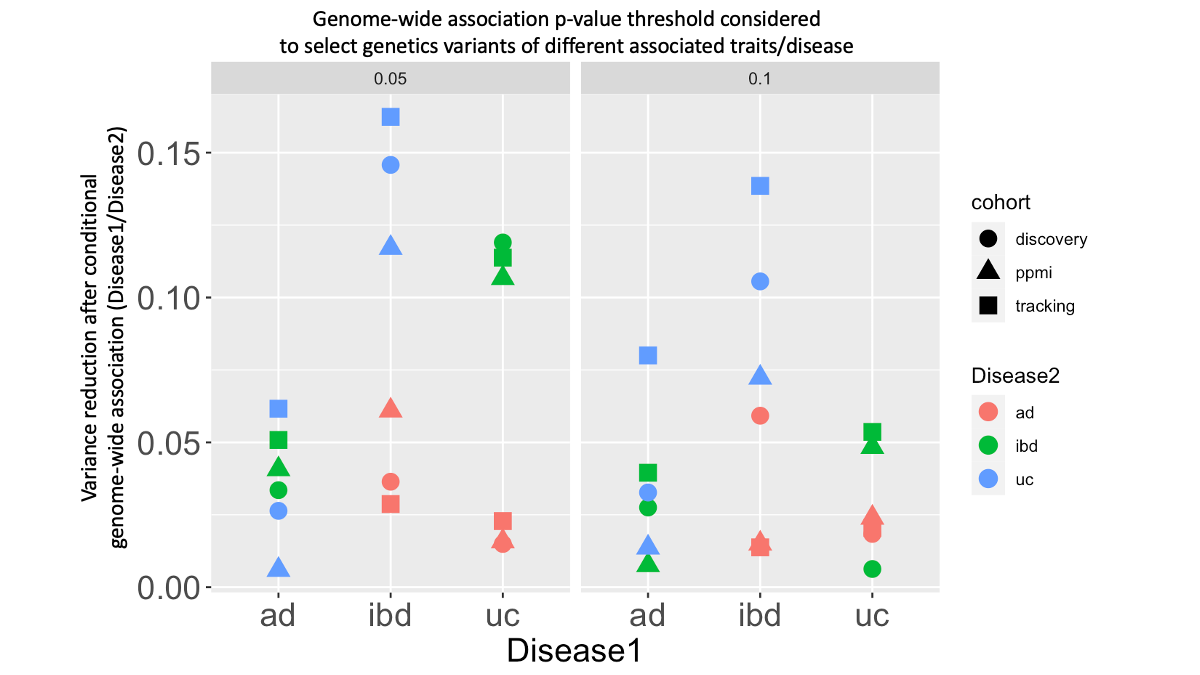
**

**Fig S16: Evaluation of the shared genetics aetiologies between pairs of disorders underlying the phenotypic axis 1.** Each plot represents the variance reduction (y) of phenotypic axis 1 calculated with the genetic risk variant of a given disorder (Disease1) after conditioning on the genetic risk of another disorder (Disease 2). The left and right plot correspond to the genetics risk variants with genetic association study of 5% and 10% respectively. The symbol represents the analyses performed in each of the three cohorts. We evaluated the following disorders: Alzheimer's disease (ad), Inflammatory Bowel Disease (ibd) and Ulcerative Colitis (uc).


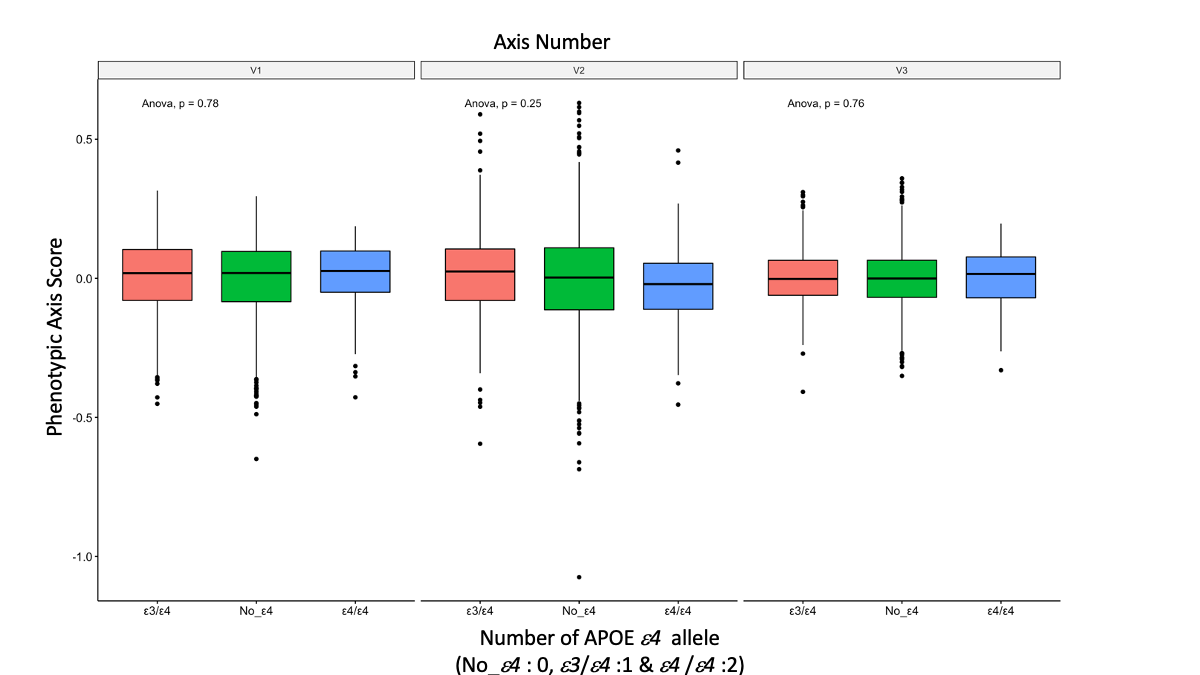


**Fig S17: No significant difference in the Parkinson’s severity symptoms measured by the phenotypic axes between individuals carrying or not carrying APOEe4 allele.** Each boxplot represents for each phenotypic axis (V1: Axis 1, V2: Axis 2, V3: Axis 3), the distribution of phenotypic axis scores according to the number of copies of APOE allele e4 carry by each individual in the *Oxford Discovery* cohort. We compared with the distribution between 3 genotype categories with ANOVA test.


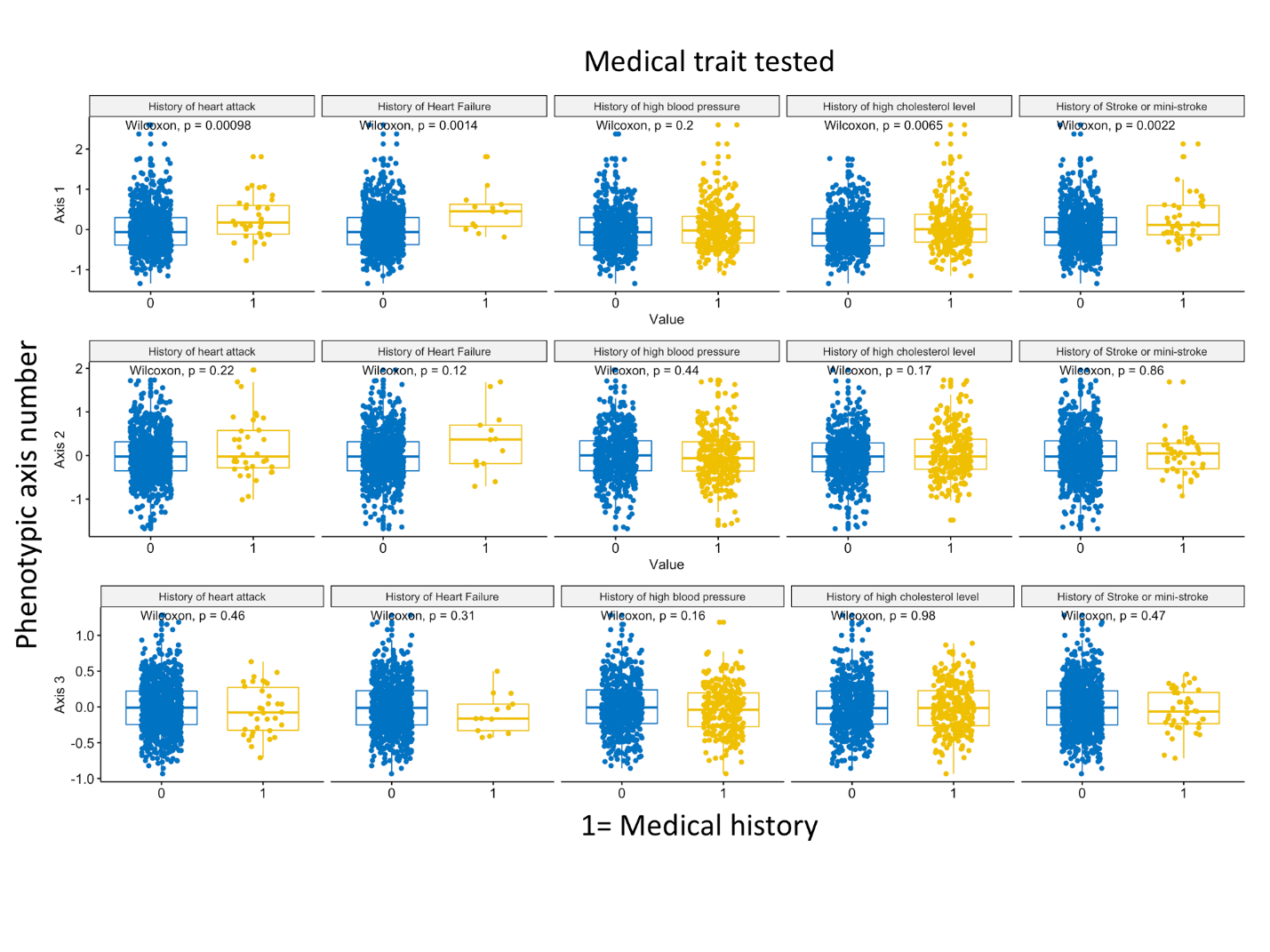


**Fig S18: Comparison of phenotypic axis score between patients in the Discovery cohorts with (=1, yellow dots) or without medical history (=0, blue dots) regarding cardio and metabolic trait.**

Each boxplot shows the distribution of phenotypic axis score according to the medical history of a Parkinson’s disease patients. We performed a Wilcoxon test to evaluate the different between groups with different medical history.

**
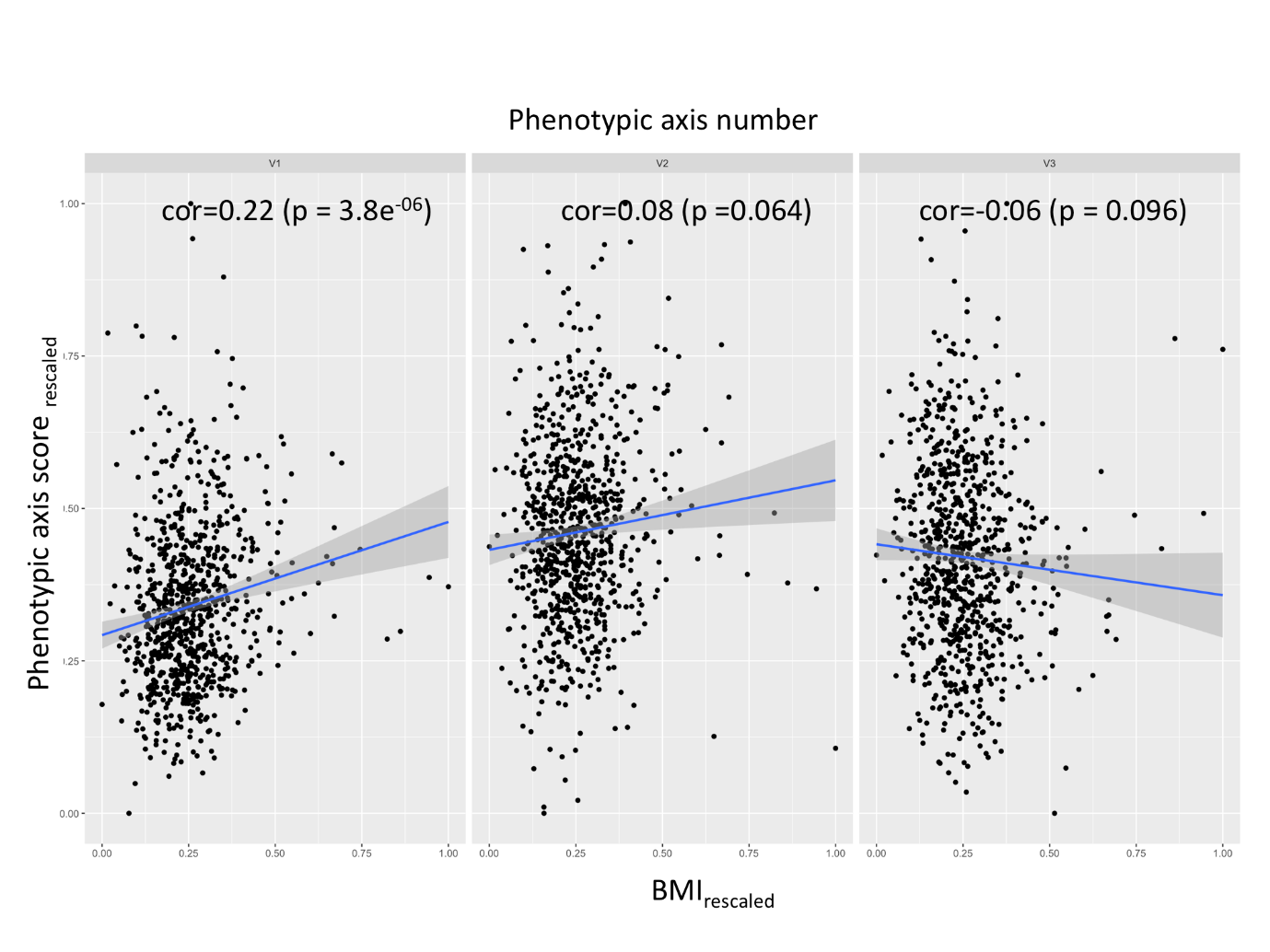
**

**Fig S19: Relation between phenotypic axis score (measure of the PD clinical variation) and the Body Mass Index in the *Discovery* cohort.**

**
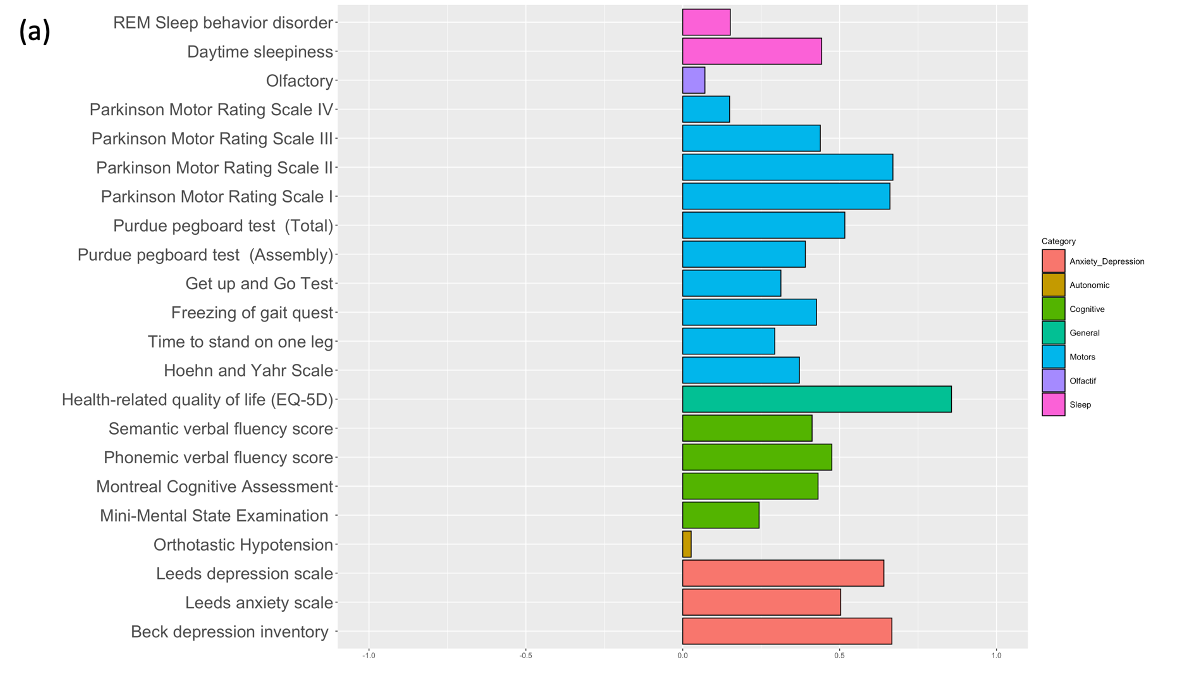
**


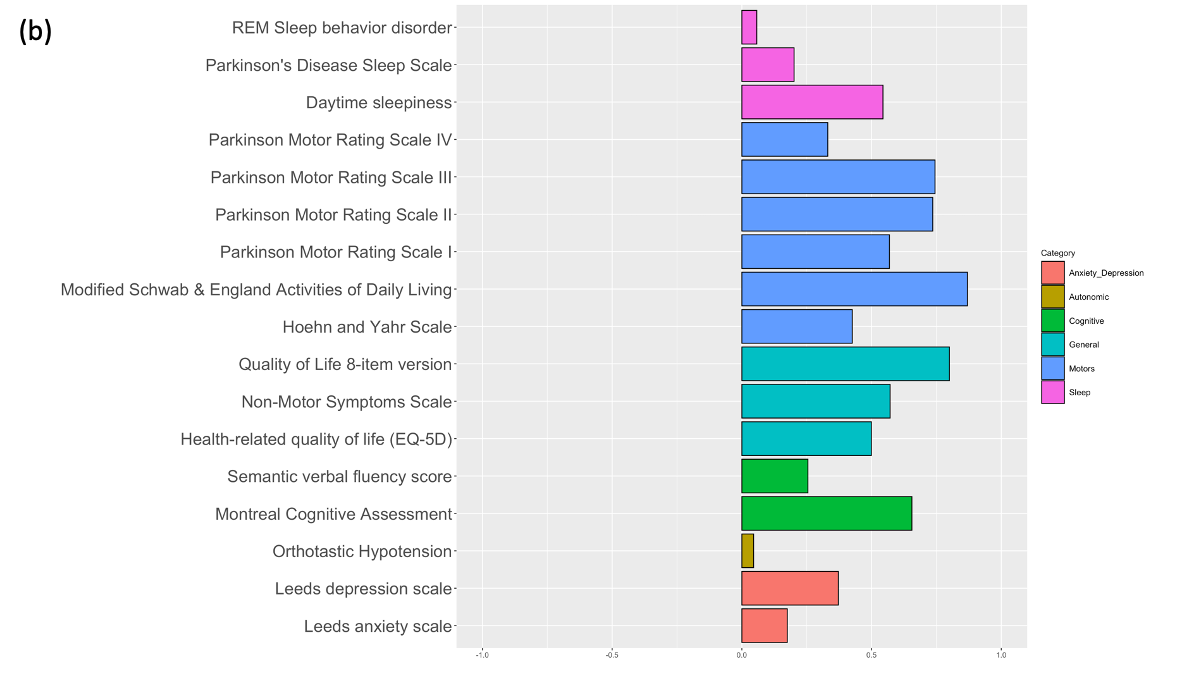


**
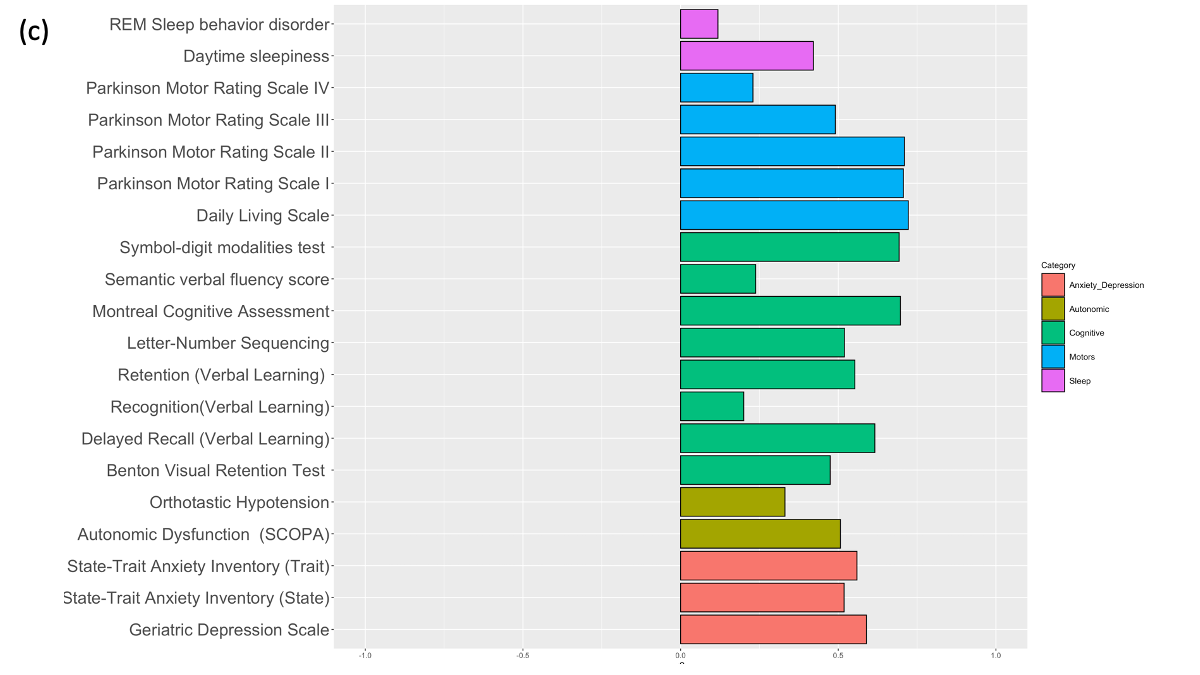
**

**Fig S20: Clinical progression phenotypes associated with the first longitudinal phenotypic axis in the *Oxford Discovery* (A), *Tracking UK* (B) and *PPMI* (C) cohort.**

Each plot represents the correlation (Pearson’s correlation coefficient r, x-axis) between the most influential longitudinal phenotypic axis and each clinical phenotypic progression measure (y-axis). The colour gradient of bars is proportional to the r coefficient.


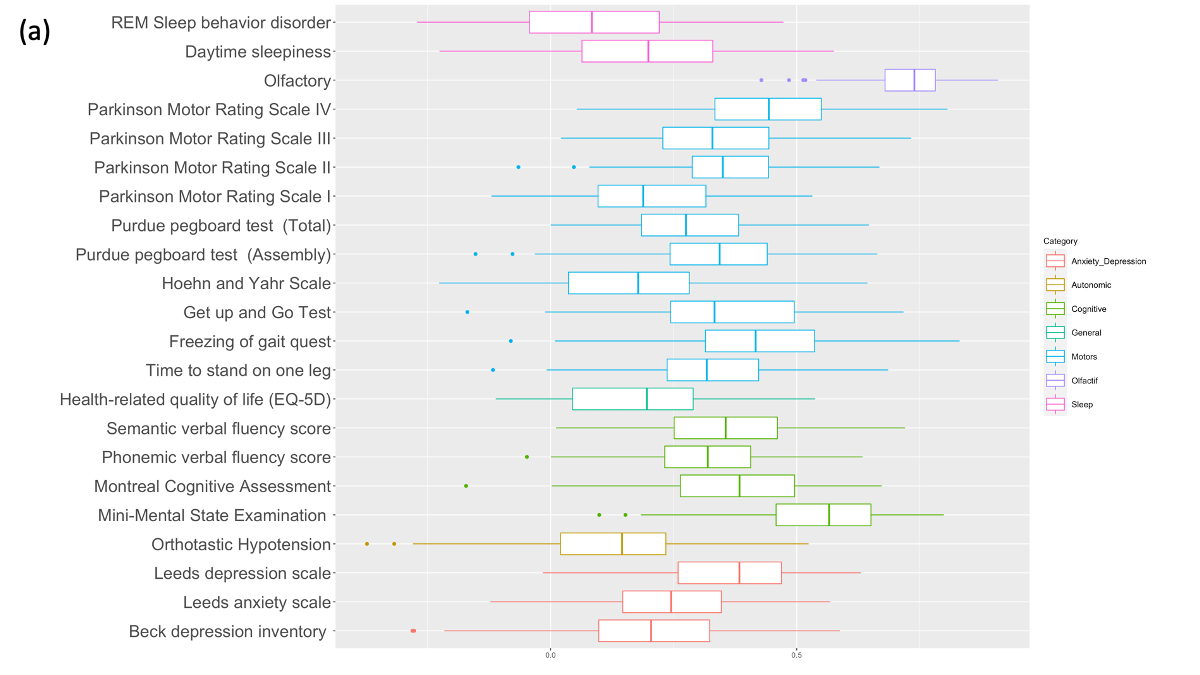


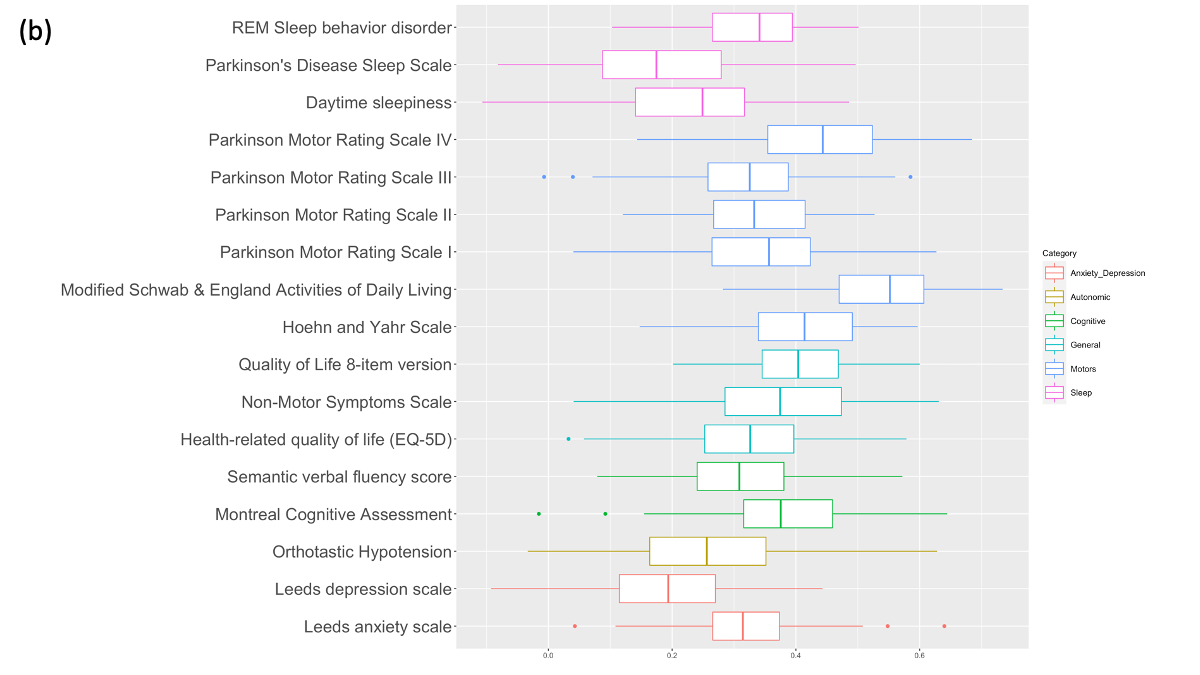


**
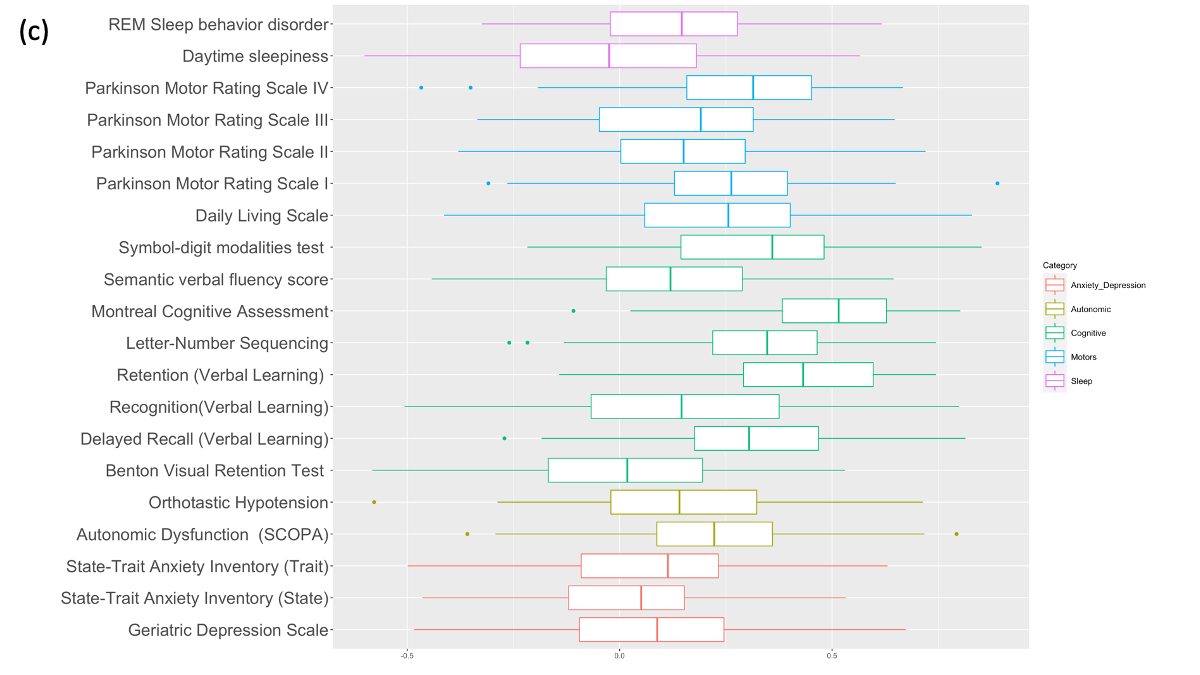
**

**Fig S21: Accuracy of the clinical course prediction in the *Oxford Discovery* (A), *Tracking* *UK* (B) and the *PPMI* cohort (C)**

Each plot represents the correlation (r2-y axis) between the imputation and the observations of clinical progression score of different phenotypes (x-axis). Here, based on the known clinical progression and the baseline clinical symptoms for 80% patients, we calculated the accuracy to predict the progression measure of a clinical phenotype given the baseline clinical features by randomly masking the clinical progression measure of 20% remaining patients.

**Fig S22: Axis 1 can predict the progression of clinical symptoms.**

Each plot represents the correlation between the Axis 1 capturing baseline clinical presentations (x-axis) and the longitudinal phenotypic axis 1 capturing the disease course in the *Oxford Discovery* (left), the *PPMI* cohort (middle) and  *Tracking UK* (right)

**
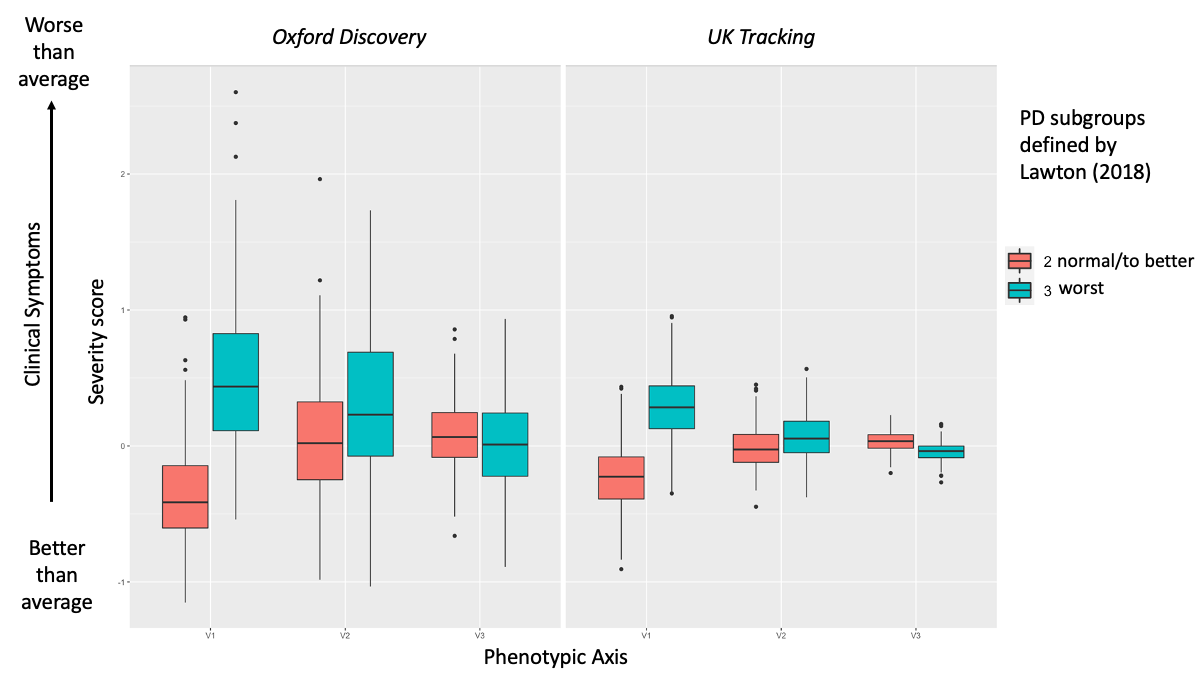
**

**Fig S23: Comparison between the phenotypic axes’ severity score and the Parkinson’s disease subgroups defined previously using a K-means clustering approach** **[12].** We examined the distribution of phenotypic axes scores (y-axis) (x-axis represents phenotypic axis number from one to three) within subgroups associated with normal/to better and worst clinical symptoms for the *Oxford Discovery* (left) and *Tracking* *UK* (right) Parkinson’s disease individuals.**
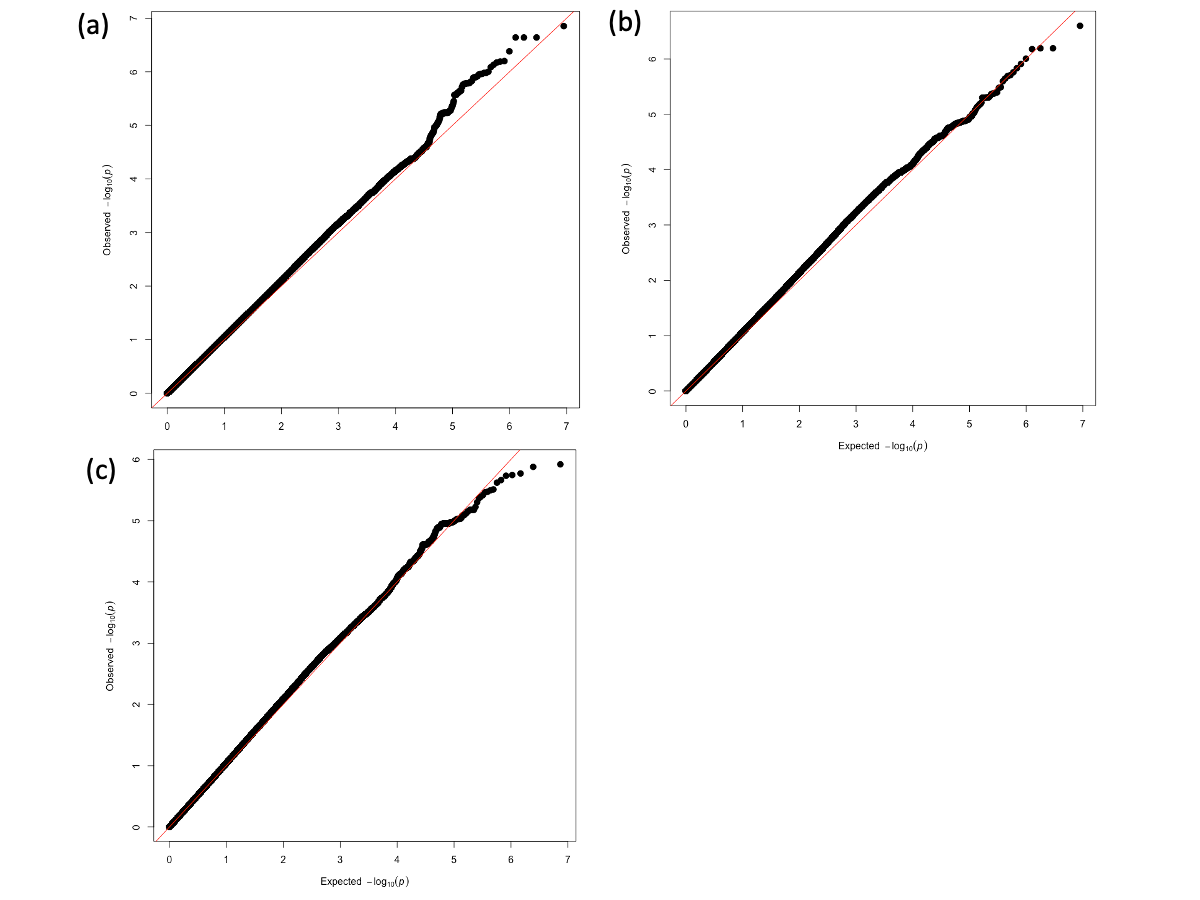
**

**Fig S24: QQ plots for the quantitative trait GWAS metanalysis** carried out for three first phenotype axis (A-C) with the summary statistic of individual GWA from 3 cohorts.


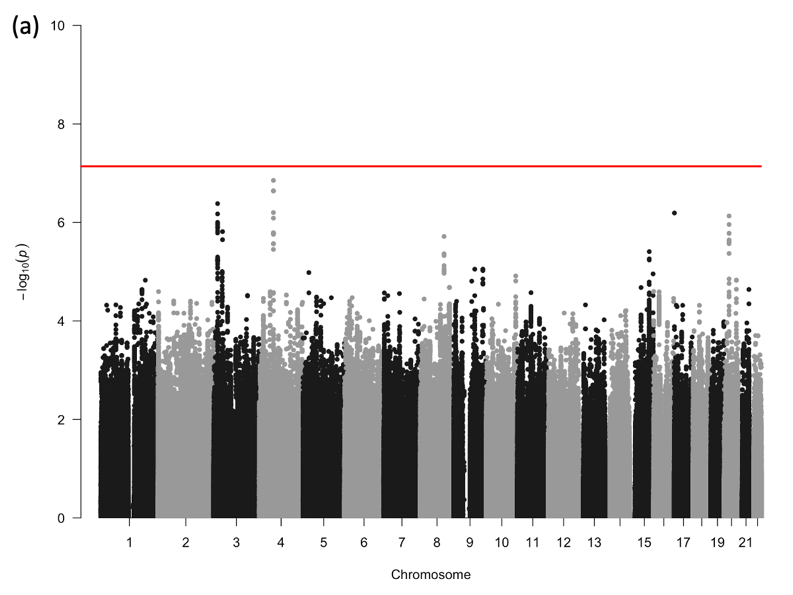


**
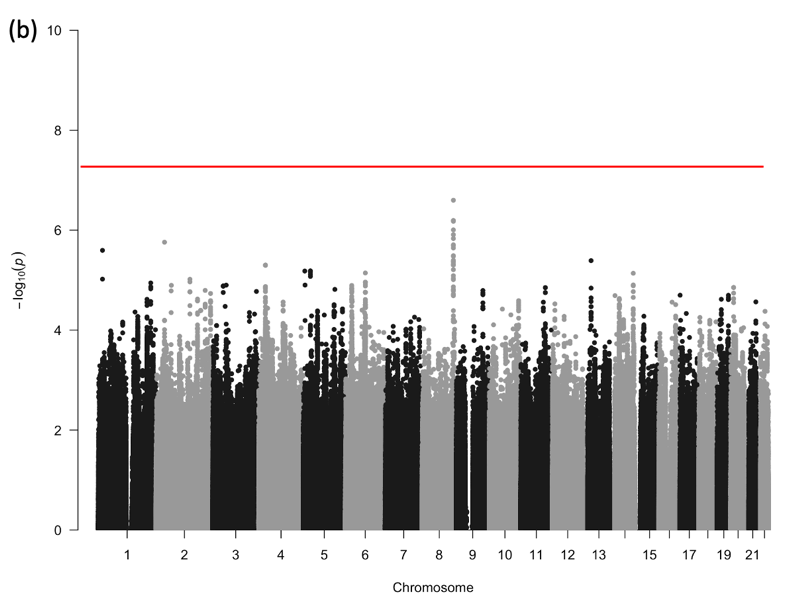
**

**
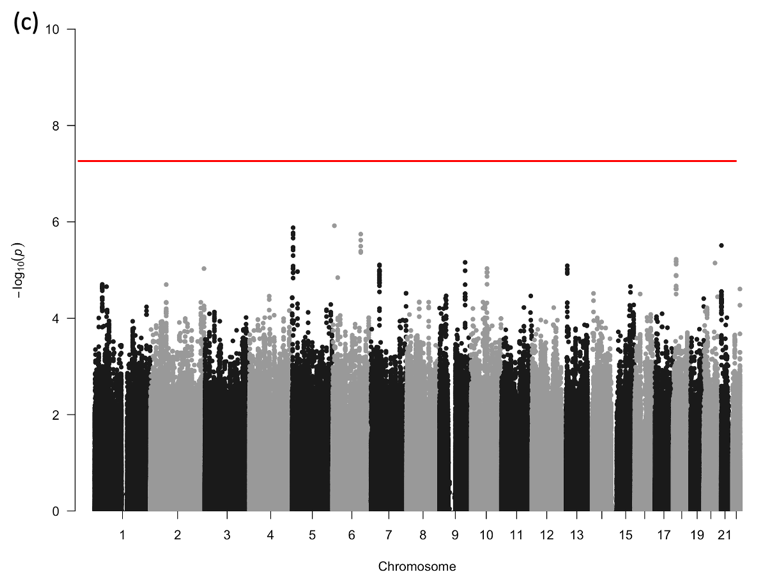
**

**Fig S25: Manhattan plots for the quantitative trait GWAS metanalysis** carried out for each phenotype axis (A-C). No variants associated with phenotypic axes superpassed genome-wide significance.

**References**

1. Purcell S, Neale B, Todd-Brown K, Thomas L, Ferreira MA, Bender D, Maller J, Sklar P, de Bakker PI, Daly MJ, Sham PC: **PLINK: a tool set for whole-genome association and population-based linkage analyses.** *Am J Hum Genet* 2007, **81:**559-575.

2. Price AL, Patterson NJ, Plenge RM, Weinblatt ME, Shadick NA, Reich D: **Principal components analysis corrects for stratification in genome-wide association studies.** *Nat Genet* 2006, **38:**904-909.

3. International HapMap C: **The International HapMap Project.** *Nature* 2003, **426:**789-796.

4. Das S, Forer L, Schonherr S, Sidore C, Locke AE, Kwong A, Vrieze SI, Chew EY, Levy S, McGue M, et al: **Next-generation genotype imputation service and methods.** *Nat Genet* 2016, **48:**1284-1287.

5. Loh PR, Danecek P, Palamara PF, Fuchsberger C, Y AR, H KF, Schoenherr S, Forer L, McCarthy S, Abecasis GR, et al: **Reference-based phasing using the Haplotype Reference Consortium panel.** *Nat Genet* 2016, **48:**1443-1448.

6. Genomes Project C, Auton A, Brooks LD, Durbin RM, Garrison EP, Kang HM, Korbel JO, Marchini JL, McCarthy S, McVean GA, Abecasis GR: **A global reference for human genetic variation.** *Nature* 2015, **526:**68-74.

7. Zhou X, Carbonetto P, Stephens M: **Polygenic modeling with bayesian sparse linear mixed models.** *PLoS Genet* 2013, **9:**e1003264.

8. Zhou X, Stephens M: **Efficient multivariate linear mixed model algorithms for genome-wide association studies.** *Nat Methods* 2014, **11:**407-409.

9. Zhou X, Stephens M: **Genome-wide efficient mixed-model analysis for association studies.** *Nat Genet* 2012, **44:**821-824.

10. Willer CJ, Li Y, Abecasis GR: **METAL: fast and efficient meta-analysis of genomewide association scans.** *Bioinformatics* 2010, **26:**2190-2191.

11. Lawton M, Baig F, Rolinski M, Ruffman C, Nithi K, May MT, Ben-Shlomo Y, Hu MT: **Parkinson's Disease Subtypes in the Oxford Parkinson Disease Centre (OPDC) Discovery Cohort.** *J Parkinsons Dis* 2015, **5:**269-279.

12. Lawton M, Ben-Shlomo Y, May MT, Baig F, Barber TR, Klein JC, Swallow DMA, Malek N, Grosset KA, Bajaj N, et al: **Developing and validating Parkinson’s disease subtypes and their motor and cognitive progression.** *Journal of Neurology, Neurosurgery & Psychiatry* 2018.
